# Genes associated with cellular senescence as diagnostic markers of major depressive disorder and their correlations with immune infiltration

**DOI:** 10.3389/fpsyt.2024.1372386

**Published:** 2024-05-31

**Authors:** Juan Chen, Xin Xie, Min Lin, Hong Han, Tingting Wang, Qirong Lei, Rongfang He

**Affiliations:** ^1^ Department of Nursing, The Affiliated Hospital of Southwest Medical University, Luzhou, China; ^2^ Department of Psychiatry, The Affiliated Hospital of Southwest Medical University, Luzhou, China; ^3^ Department of Nephrology, The Affiliated Hospital of Southwest Medical University, Luzhou, China; ^4^ Sichuan Clinical Research Center for Nephropathy, The Affiliated Hospital of Southwest Medical University, Luzhou, China; ^5^ Department of Dermatology, The Affiliated Hospital of Southwest Medical University, Luzhou, Sichuan, China

**Keywords:** depression, cellular senescence, immune infiltration, biomarkers, RNA sequecing

## Abstract

**Background:**

Emerging evidence links cellular senescence to the pathogenesis of major depressive disorder (MDD), a life-threatening and debilitating mental illness. However, the roles of cellular senescence-related genes in MDD are largely unknown and were investigated in this study using a comprehensive analysis.

**Methods:**

Peripheral blood microarray sequencing data were downloaded from Gene Expression Omnibus (GEO) database and retrieved cellular senescence-related genes from CellAge database. A weighted gene co-expression network analysis was used to screen MDD-associated genes. Protein-protein interactions (PPI) were predicted based on STRING data, and four topological algorithms were used to identify hub genes from the PPI network. Immune infiltration was evaluated using CIBERSORT, followed by a correlation analysis between hub genes and immune cells.

**Results:**

A total of 84 cell senescence-related genes were differentially expressed in patients with MDD compared to healthy control participants. Among the 84 genes, 20 were identified to be associated with the MDD disease phenotype, and these genes were mainly involved in hormone-related signaling pathways (such as estrogen, steroid hormone, and corticosteroid) and immune and inflammatory pathways. Three genes, namely, JUN, CTSD, and CALR, which were downregulated in MDD, were identified as the hub genes. The expression of hub genes significantly moderate correlated with multiple immune cells, such as Tregs, NK cells, and CD4+ T cells, and the abundance of these immune cells markedly differed in MDD samples. Multiple microRNAs, transcription factors, and small-molecule drugs targeting hub genes were predicted to explore their molecular regulatory mechanisms and potential therapeutic value in MDD.

**Conclusion:**

JUN, CTSD, and CALR were identified as potential diagnostic markers of MDD and may be involved in the immunoinflammatory mechanism of MDD.

## Highlights

- Peripheral nervous system myelin maintenance was inactivated in patients with MDD.
- Three potential diagnostic markers, JUN, CTSD, and CALR were identified in MDD.
- Expression of marker genes correlated with immune cells infiltration.

## Introduction

Major depressive disorder (MDD) is a common mental disorder characterized by a significant and persistent low mood, loss of interest, sleep disturbances, and suicidality, severely diminishing psychosocial functioning and the quality of life of patients (1). Although the prevalence of MDD varies among different populations, the overall 12-month and lifetime prevalences are approximately 6-10% and 15-20%, respectively (2–4). Also, MDD is a recurrent lifelong illness and is ranked as a leading cause of disability worldwide and is a highly heterogeneous and multifactorial disorder (5–7). Its diagnosis mainly relies on the subjective identification of patient symptoms by psychiatrists and depression rating scales, which results in a considerable misdiagnosis rate and limited effective treatment (3). Although multiple biomarkers for MDD, such as neuroendocrine hormones, neurotransmitters, and markers involved in inflammatory and metabolic processes, have been proposed over the past few decades, their application in a clinical context has been limited because of their unsatisfactory sensitivity and specificity (8–10). Additionally, current treatments are ineffective in 20-40% of patients (11), and novel drug development strategies are required to address this issue, thus highlighting the importance of uncovering the pathophysiological basis and identifying objective diagnostic biomarkers (12).

Cellular senescence is a state of irreversible growth arrest resulting from various stresses such as telomere attrition, oxidative stress, and replicative exhaustion (13). A molecular intersection between cell senescence and MDD has been demonstrated. Mendes-Silva et al. (14) revealed that in the context of late-life depression, senescence-associated secretory phenotype proteins are associated with microstructural abnormalities in white matter tracts in brain and worse executive function performance. Diniz et al. (15) have found that molecular and cellular senescence, as measured with the senescence-associated secretory phenotype index, is associated with worse treatment outcomes in late-life depression. Telomere shortening, a hallmark of cellular senescence, occurs more frequently in patients with MDD than in those without MDD, especially in those with a more severe depressive episode (16), and a high severity of depressive symptoms was linked to a short average leukocyte telomere length in a 10-year longitudinal study (17). Overexpression of cell cycle regulator markers p21 is typical feature of senescent cells (18, 19). Markedly elevated p21 expression in hippocampal neurons and leukocytes was observed in patients with MDD and was directly correlated with depression and anxiety scores (20, 21). Ogrodnik et al. (22) reported that clearance of senescent cells from obese mice could alleviate anxiety-like behaviors, indicating the potential therapeutic value of senolytics in neuropsychiatric diseases. Additionally, a high senescence-associated secretory phenotype index has been proposed as a biomarker of MDD episodes (23). These findings emphasize the involvement of cellular senescence in the pathophysiology of MDD. Nevertheless, cellular senescence-related biomarkers and therapeutic targets for MDD have not been fully investigated.

Mounting evidence has demonstrated different microRNAs (miRNAs) involvement in the set of diverse pathways associated with MDD, including the miR-144-5p, miR-155, miR-146a (24, 25). Besides, transcription factors (TFs) recognize specific DNA sequences to control chromatin and transcription, forming a complex system that directs genome expression (26). Fan et al. (27) illustrated that alterations in TF Phox2 expression may play a role in MDD and possibly other stress-related disorders through their modulatory effects on the noradrenergic phenotype. Consequently, in this study, a comprehensive transcriptomic data analysis was conducted to elucidate the biological patterns of cellular senescence-related genes in MDD. The most potent genes associated with cell senescence and MDD were identified using weighted gene co-expression network analysis (WGCNA) and multiple topology analysis algorithms. The association of these genes with immune infiltration, molecular regulatory networks, and small-molecule drugs targeting these genes were further explored. Overall, this study provides novel insights into the pathophysiology of MDD and helps identify promising biomarkers or therapeutic targets.

## Methods

### Data acquisition and preprocessing

The microarray datasets GSE201332 and GSE52790 were downloaded from the Gene Expression Omnibus (GEO) database. The GSE201332 dataset comprises of the data from 40 peripheral blood samples obtained from 20 patients with MDD and 20 healthy control participants using the GPL3219 Agilent-052909 platform. Data from the GSE52790 dataset were sequenced on GPL17976 platform, which included 22 peripheral blood samples obtained from 10 patients with MDD and 12 healthy control participants. The probes were annotated with the respective gene symbols based on the annotation data of the sequencing platform, and those that had no corresponding gene symbol were discarded. When multiple probes matched the same gene symbol, the gene expression value was calculated as the mean value. This study was conducted using two independent datasets, the GSE201332 dataset for discovery and the GSE52790 for validating the results. The demographic and clinical characteristics of the individuals included in the GSE201332 and GSE52790 datasets were illustrated in **Supplementary Materials 1** and **2**, respectively.

### Differentially expressed cell senescence-related genes

Senescence-related genes were obtained from the CellAge Database. Expression data for these genes were extracted from the GSE201332 dataset, followed by differential expression analysis between the MDD and control groups using the limma package (version 3.34.7). Differentially expressed senescence-related genes were screened based on the criterion of false discovery rate (FDR) < 0.05.

### WGCNA

The top 20% of genes with large variations between the MDD and control samples were analyzed using WGCNA (version 1.71) to identify MDD-associated gene modules. Briefly, an unsupervised co-expression matrix was defined, and a soft-threshold power (the square value of the correlation coefficient that reaches 0.85 for the first time) was selected to balance scale-free networks. Next, a hierarchical gene clustering dendrogram and dynamic tree cutting were conducted to identify highly correlated gene modules with a minModuleSize setting of 30. Finally, the correlations between each gene module and clinical traits (MDD disease phenotype and normal) were calculated to determine the gene modules that were significantly related to MDD, with cut-off values of p < 0.01 and |r| > 0.8.

### Genes associated with both cell senescence and MDD

Venn analysis was performed to identify genes shared between the MDD-associated gene modules and differentially expressed senescence-related genes using the VennDetail package (version 1.2.0). These shared genes were regarded as genes associated with both senescence and MDD. Enrichment analysis for gene ontology biological processes (BP) terms and KEGG pathways were conducted using clusterProfiler (version:3.16.0), and the significantly enriched results were selected based on the criteria of adjusted p < 0.05 and count ≥ 2. Interactions among proteins encoded by the genes were predicted based on data from the STRING (version 12) database, with the protein-protein interaction (PPI) score set at 0.15, and the PPI network was visualized using Cytoscape (version 3.6.1).

### Identification of hub genes

Topological algorithms, including maximal clique centrality (MCC), maximum neighborhood component (MNC), degree, and edge-percolated component (EPC) of the CytoHubba plug-in were used to screen the top 10 genes from the PPI network, and the overlapping genes were identified as hub genes. To validate these identified hub genes, the expression of hub genes in the GSE52790 validation dataset was explored, and the difference between the expression values of the hub genes in patients with MDD and control participants was analyzed using t-tests.

### Gene set variation analysis

With the BP terms and KEGG pathway gene sets in MsigDB database as the enrichment background, the enrichment scores of all KEGG pathways and BP terms in each sample were calculated using GSVA (version 1.36.2) to generate a scoring matrix. Differences between the enrichment scores of MDD and control samples were calculated using the limma package, with cut-off values of |logFC| > 2 and adjusted p < 0.05.

### Gene set enrichment analysis

With the gene sets of KEGG pathways, hallmark genes, and BP terms in MsigDB database used as enrichment background, and GSEA was performed using clusterProfiler package (version 4.4.4). A threshold of adjusted p < 0.05 was used to select the significantly enriched results.

### Correlation between hub genes and immune status

Based on the gene expression profiles, the infiltration of diverse immune cells in MDD and control samples was inferred using the CIBERSORT algorithm. The infiltration proportion of each immune cell type, expression of immune checkpoint genes, and expression of human leukocyte antigen (HLA) genes between the MDD and control groups were compared using the Wilcoxon test. Spearman correlations of hub gene expression with immune cell infiltration, immune checkpoint genes, and HLA genes were explored using the COR function.

### Construction of regulatory networks

MiRNAs and TFs that may target hub genes were predicted using the ENCORI tool and TRRUST database, respectively. miRNA–gene pairs reported commonly in at least three databases were selected for network construction. Regulatory networks were constructed based on the predicted miRNA-gene and TF-gene pairs.

### Prediction of small-molecule drugs

Drug-gene relationship pairs were predicted for hub genes based on the Drug-Gene Interaction database (DGIdb, http://dgidb.org/), with a search scope of 20 commonly used drug databases. The drug-gene network was visualized using Cytoscape (version 3.6.1).

## Results

### Analysis of altered signaling pathways in MDD

**Figure 1:**
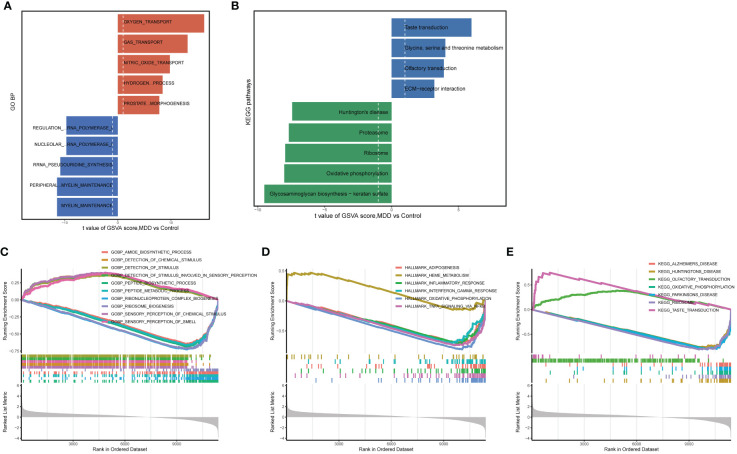
Enrichment analysis for identifying altered functional pathways in MDD. **(A, B)** Significantly altered gene ontology biological processes **(A)** and KEGG pathways **(B)** between major depressive disorder (MDD) and control group samples analyzed by gene set variation analysis (GSVA); **(C–E)** significantly altered gene ontology biological processes **(C)**, hallmark genes **(D)**, and KEGG pathways **(E)** between MDD and control group samples analyzed by gene set enrichment analysis (GSEA).

A preliminary analysis of the data from patients with MDD and healthy control participants was conducted. Large-scale changes in functional pathways were observed in the MDD group compared to those in the healthy participants. Total 59 markedly altered KEGG pathways and 1190 BP terms were obtained using GSVA. In terms of BP annotations, the transport of oxygen, gas, nitric oxide, and hydrogen peroxide catabolic processes were activated, whereas peripheral nervous system myelin maintenance was inactivated in MDD (**Figure 1A**). In terms of KEGG pathways, taste transduction; olfactory transduction; and glycine, serine and threonine metabolism were activated, whereas Huntington’s disease, oxidative phosphorylation, and glycosaminoglycan biosynthesis-keratan sulfate were inactivated in patients with MDD compared to those in healthy participants (**Figure 1B**). Consistently, GSEA revealed 55 markedly dysregulated KEGG pathways, 24 hallmarks, and 1223 BP annotations. For example, BP annotation terms such as amide biosynthetic process, sensory perception of chemical stimulus and smell, detection of chemical stimulus, and detection of stimulus involved in sensory perception were dysregulated in patients with MDD (**Figure 1C**). In addition, the hallmarkinterferon gamma response, hallmark_infilammatory response, hallmark_oxidative phosphorylation, KEGG pathways associated with neurodegenerative diseases (Alzheimer’s disease, Huntington’s disease, Parkinson’s disease), olfactory and taste transduction, and oxidative phosphorylation were altered in patients with MDD compared those in healthy participants (**Figures 1D, E**). These altered functional pathways may be important in the pathophysiological mechanisms of MDD.

### Dysregulation of cell senescence-related genes in MDD

**Figure 2:**
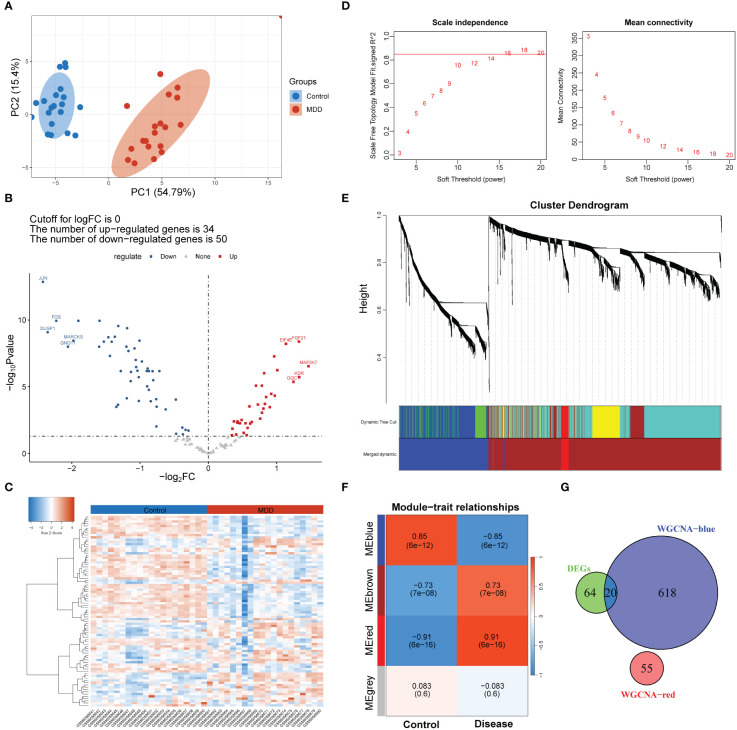
Screening of genes associated with both MDD and cell senescence. **(A)** Principal component analysis of MDD and control group samples; **(B, C)** Volcano plot **(B)** and heatmap **(C)** showing the number and expression pattern of differentially expressed cell senescence-related genes between MDD and control groups; **(D)** Calculation of soft threshold (power) for weighted gene co-expression network analysis (WGCNA); **(E)** Cluster dendrogram generated by hierarchical clustering based on the dissimilarity measure of genes; **(F)** Heatmaps of module-trait relationships; **(G)** Venn diagram identifying 20 shared genes between the differentially expressed genes and WGCNA module genes.

A differential analysis of 127 senescence-related genes expressed in the GSE201332 database was conducted between patients with MDD and healthy participants. Principal component analysis confirmed the differences between the two sample groups (**Figure 2A**). Subsequently, 84 dysregulated senescence-related genes were revealed in MDD samples compared to healthy participants (**Figure 2B**). A heatmap verified the diverse expression patterns of these genes between groups (**Figure 2C**), indicating a possible link between cell senescence and MDD.

### Screening of genes associated with both MDD and cell senescence

**Figure 3:**
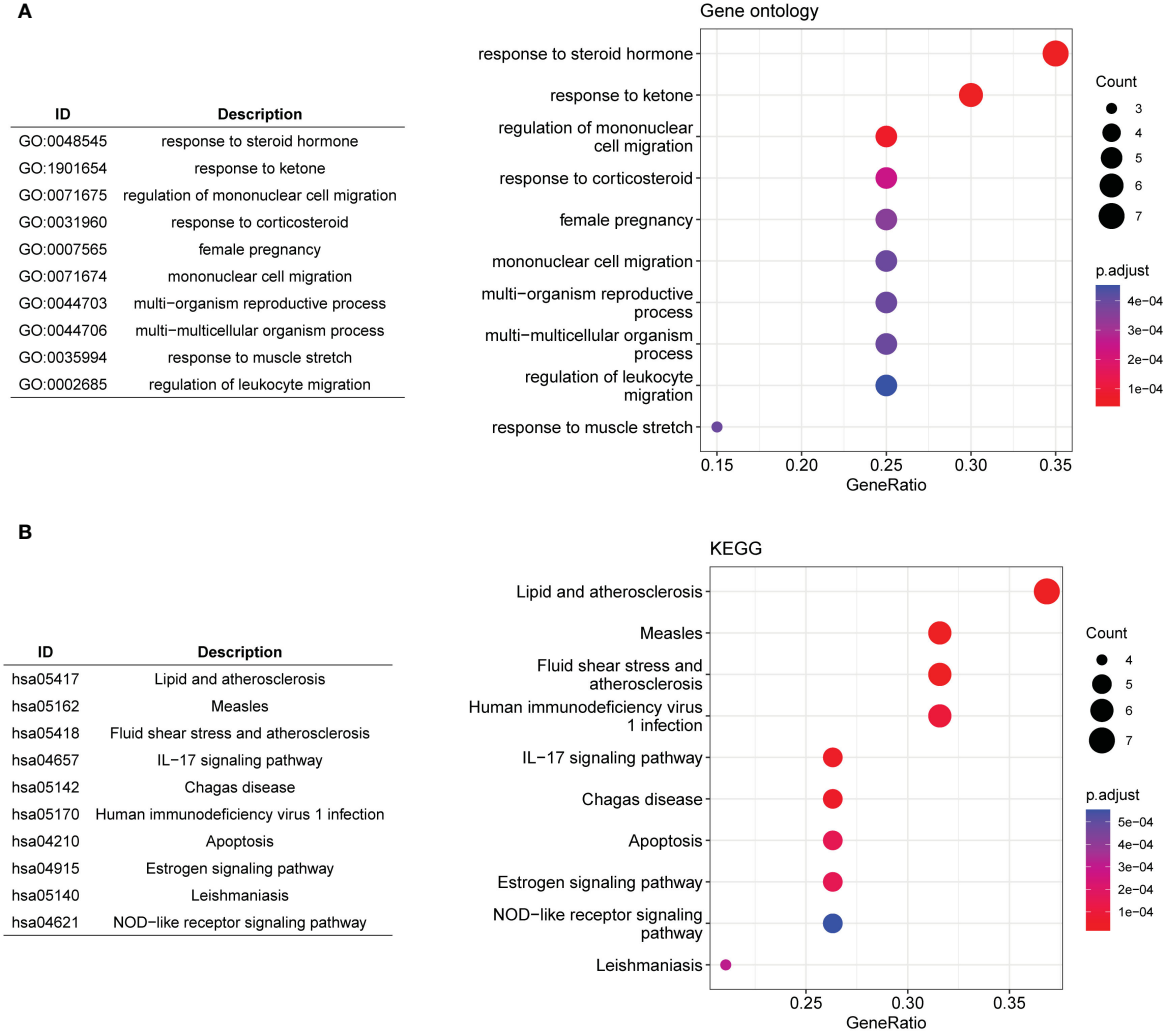
Functional enrichment analysis. **(A, B)** The top 10 significantly enriched gene ontology biological processes **(A)** and KEGG pathways **(B)** for the 20 shared genes.

A scale-free network was constructed (**Figure 2D**), and a total of four gene modules were identified, and heatmap of module–trait relationships showed that “blue” (r = -0.85, p =0.000) and “red” modules (r = 0.91, p = 0.000) were markedly high correlated with disease phenotype of MDD (**Figures 2E, F**). Venn analysis identified 20 shared genes among dysregulated senescence-related and MDD-associated module genes (**Figure 2G**), which were defined as genes associated with both MDD and senescence and were used for subsequent analysis. These genes were significantly involved in the response to steroid hormones, response to corticosteroids, and regulation of migration of mononuclear cells, such as leukocytes (**Figure 3A**). In addition, KEGG enrichment analysis revealed that they were mainly implicated in the estrogen signaling pathway, IL−17 signaling pathway, and atherosclerosis-related pathways, such as lipid and atherosclerosis, fluid shear stress, and atherosclerosis (**Figure 3B**).

### Identification of hub genes

**Figure 4:**
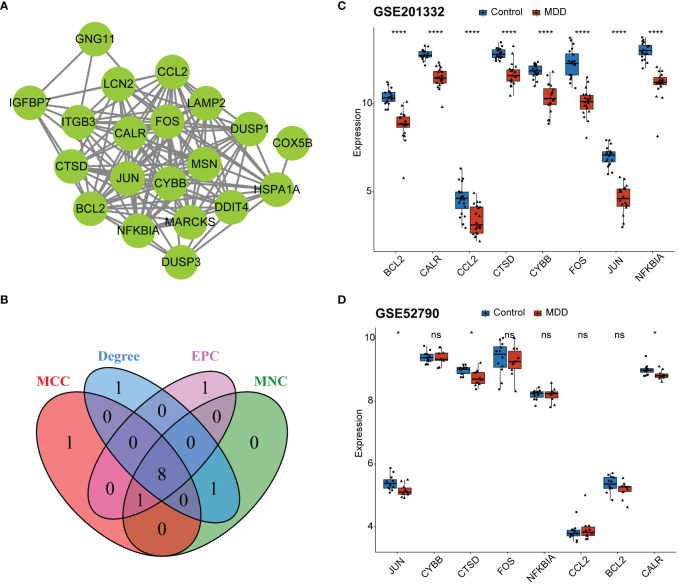
Identification of hub genes. **(A)** Protein-protein interaction (PPI) network for the 20 shared genes; **(B)** Venn diagram identifying eight key genes from the PPI network based on four topological algorithms; **(C)** boxplot showing expression of eight key genes between MDD and control samples in the GSE201332 training dataset; **(D)** boxplot showing validation of eight key gene expression using the GSE52790 independent dataset. *p < 0.05; ****p < 0.0001; ns, no statistical significance.

PPI network was constructed on the 20 genes associated with MDD and senescence (**Figure 4A**). The nodes in the PPI network were tightly linked, indicating close interactions among these genes. The top 10 genes were screened using four topological algorithms, which revealed 8 shared genes (**Figure 4B**), including JUN, CYBB, CTSD, FOS, NFKBIA, CCL2, BCL2 and CALR. All these genes were downregulated in patients with MDD compared to healthy controls (**Figure 4C**). The expression of these genes was validated in GSE52790 validation dataset (**Figure 4D**). Consistently, JUN, CTSD, and CALR were downregulated in MDD, whereas the expression of other genes showed no significant differences between MDD and control samples. Therefore, JUN, CTSD, and CALR were selected as hub genes for subsequent analyses.

### Correlation of hub gene expression with immune status

**Figure 5:**
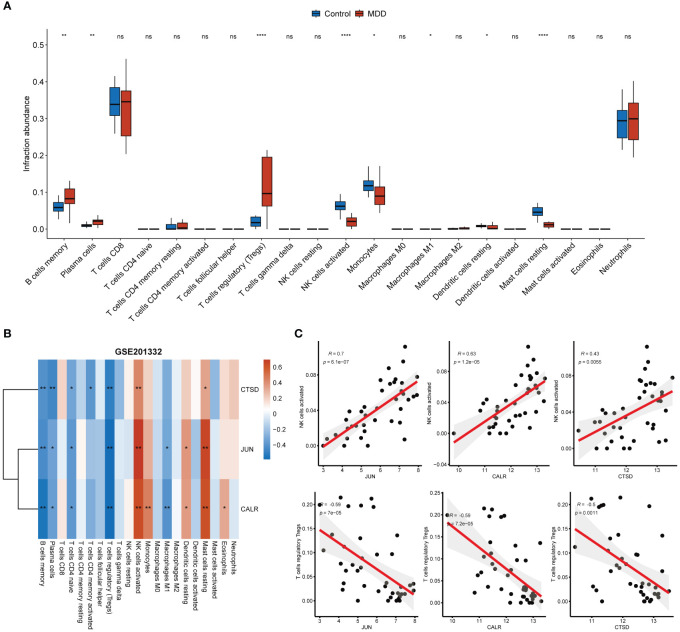
Associations of hub genes with immune infiltration. **(A)** Boxplot showing the differences in immune cell infiltration abundance between MDD and control samples; **(B)** Correlation heatmap showing the correlations between hub gene expression and immune cell infiltration abundance; **(C)** Correlation scatter plot showing the strong positive correlations between hub gene expression and the infiltration abundance of activated NK cells and the strong negative correlations between hub gene expression and the infiltration abundance of Tregs. *p < 0.05; **p < 0.01; ****p < 0.0001; ns, no statistical significance.

**Figure 6:**
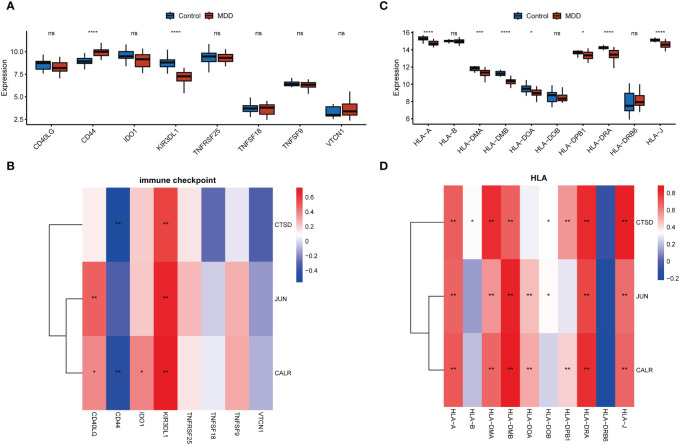
Associations of hub genes with immune checkpoints and HLA genes. **(A, C)** Boxplots showing the differences in the expression of immune checkpoint genes **(A)** and HLA genes **(C)** between the MDD and control groups; **(C D)**, Correlation heatmaps showing the correlations of hub gene expression with immune checkpoint genes **(B)** and HLA genes **(D)**. *p < 0.05; **p < 0.01; ***p < 0.001; ****p < 0.0001; ns, no statistical significance.

CD8+ T cells and neutrophils were found to have an obviously higher proportion than other immune cells in both MDD and control samples (**Figure 5A**). The infiltration of seven types of immune cells, including memory B cells, plasma cells, regulatory T cells (Tregs), activated NK cells, monocytes, M1 macrophages, resting dendritic cells, and resting mast cells, differed between the MDD and control samples (**Figure 5A**). In particular, a high proportion of Tregs and memory B cells and a low proportion of activated NK cells, monocytes, and resting mast cells were found in MDD samples. All three hub genes significantly correlated with the infiltration of multiple types of immune cells (**Figure 5B**). Specifically, all three hub genes showed moderate positive correlations (r = 0.7 for JUN, r = 0.63 for CALR, r = 0.43 for CTSD, all p < 0.05) with activated NK cells and showed moderate negative correlations (r = −0.59 for JUN, r = −0.59 for CALR, r = −0.50 for CTSD, all p < 0.05) with Tergs (**Figure 5C**). In addition to immune cell infiltration, the expression of immune checkpoint and HLA genes was evaluated (**Figure 6**). Only the immune checkpoints CD44 and KIR3DL1 were dysregulated (**Figure 6A**), and all three hub genes were positively correlated with KIR3DL1 expression (**Figure 6B**). Interestingly, the expression of multiple HLA genes was reduced in MDD samples compared to that in control samples (**Figure 6C**), and these dysregulated HLA genes were positively correlated with hub genes (**Figure 6D**). These findings demonstrate that these three hub genes may be involved in regulating the immune status of patients with MDD.

### Regulatory networks for hub genes

**Figure 7:**
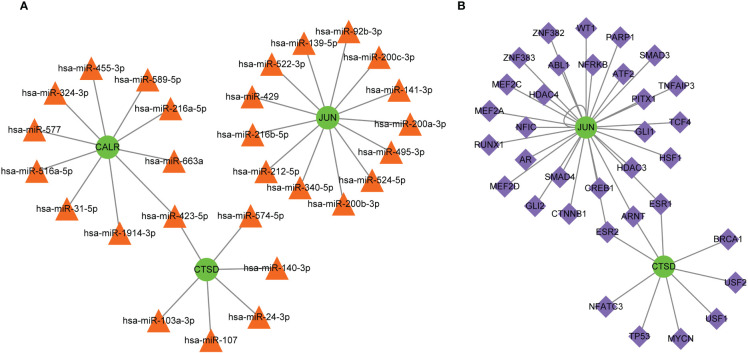
Molecular regulatory networks for hub genes. **(A)** The miRNA-gene regulatory network, in which green circles represent hub genes and orange triangles represent miRNAs; **(B)** the transcription factor-gene regulatory network, in which green circles represent hub genes and purple diamonds represent transcription factors.

**Figure 8:**
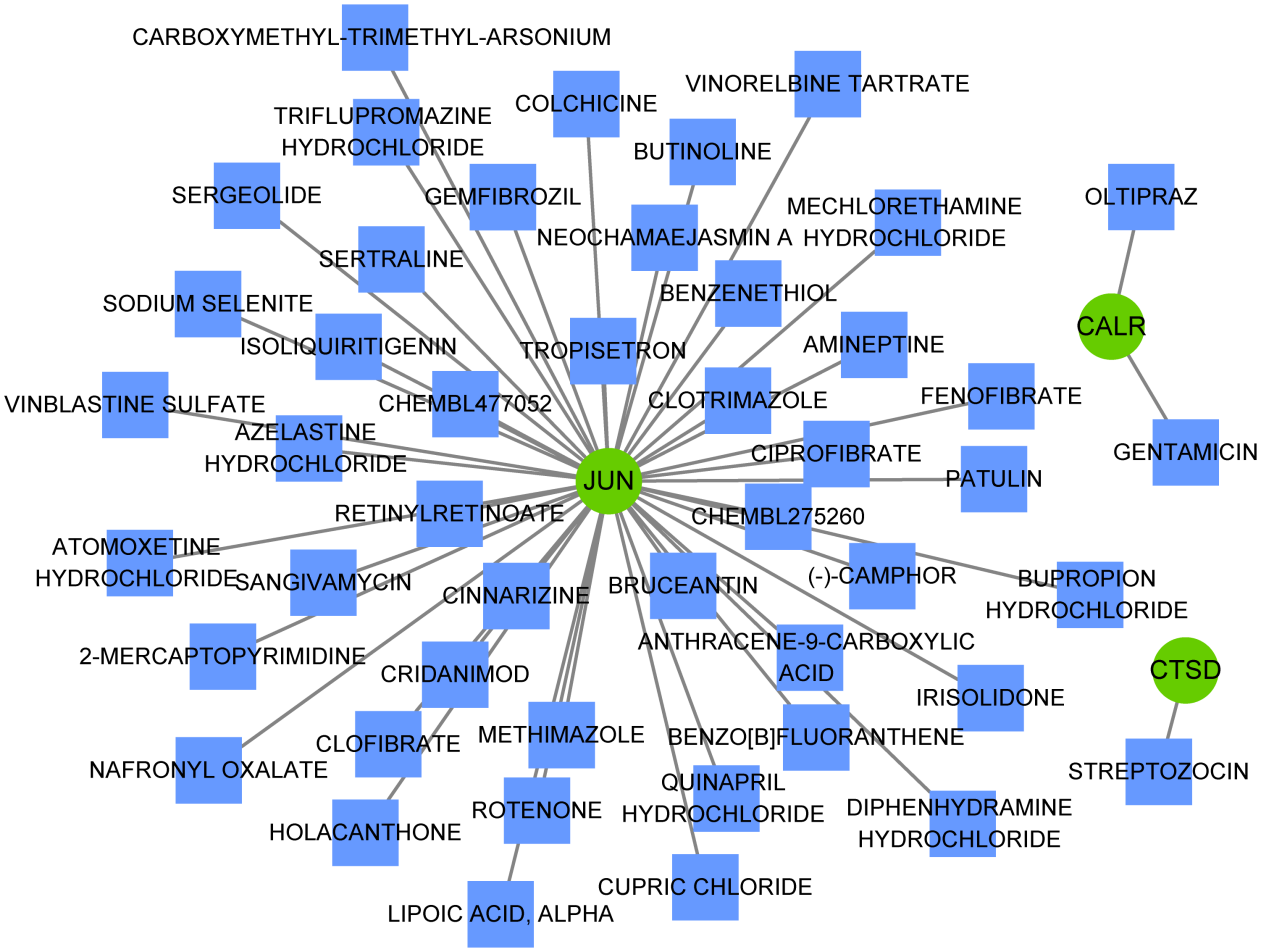
Small-molecule drugs targeting hub genes. Small-molecule drugs targeting hub genes were analyzed using the Drug-Gene Interaction database. Green circles represent hub genes and blue squares represent small-molecule drugs.

Multiple miRNAs were found to target these three hub genes, with 13 miRNAs targeting JUN, 10 miRNAs targeting CALR, and 6 miRNAs targeting CTSD (**Figure 7A**). For example, CTSD and CALR are the potential targets of miR-423-5p. In addition, JUN and CTSD, particularly JUN, were regulated by multiple TFs (**Figure 7B**). ESR1, ESR2, and ARNT are TFs that regulate JUN and CTSD. Multiple miRNAs and TFs have been predicted to target these genes, indirectly demonstrating the importance of hub genes. Small-molecule drug predictions were conducted based on these hub genes (**Figure 8**). CTSD is a target of streptozocin, and CALR is the target of oltipraz and gentamicin, whereas JUN is a target of more than 40 drugs.

## Discussion

To our knowledge, MDD is a common clinical mental disorder affecting over 350 million people worldwide (28), which has a high rate of misdiagnosis owing to its high heterogeneity and complex pathophysiology. Moreover, conventional treatments are ineffective in a significant proportion of patients. Successful novel drug development strategies are limited by the lack of a pathophysiological basis, objective diagnostic biomarkers, and translational animal models (12). Emerging evidence has linked cellular senescence to the pathogenesis of depression (23, 29, 30). In this study, a comprehensive analysis was conducted to elucidate the biological patterns of cellular senescence-related genes and identify novel biomarkers and drug targets in MDD. The results showed that total 20 were identified associated with the MDD disease phenotype, and these genes were mainly involved in hormone-related signaling pathways and immune and inflammatory pathways. Three genes, namely, JUN, CTSD, and CALR, were identified as the hub genes. The expression of hub genes significantly moderate correlated with multiple immune cells, such as Tregs, NK cells, and CD4+ T cells, and the abundance of these immune cells markedly differed in MDD samples.

To gain a more comprehensive understanding of the pathophysiological basis of MDD, the signaling pathways that markedly dysregulated in MDD were first analyzed, using GSEA and GSVA. In this analysis, the inactivation of peripheral nervous system myelin maintenance was observed in patients with MDD. Myelin is a lipid wrapped around the outside of the nerve axon that provides protection and insulation. Myelin can be degraded when the central nervous system (CNS) malfunctions, which may damage the transmission of neural signals, thereby impairing normal physiological functions (31). Myelin deficits/decreases are involved in the pathophysiology of MDD (32), and myelin deficits in the fornix are cumulative with disease progression (33). Along with myelin maintenance inactivation, changes in sensory perception transduction, such as taste and smell, and signaling pathways/hallmarks related to neurodegenerative diseases were also observed in patients with MDD compared those in healthy controls. Dysregulation of inflammation-related pathways or hallmarks, known processes implicated in the pathophysiology of MDD, has also been observed. There appears to be a bidirectional association between the inflammatory processes and MDD (34). Inflammation is a key disease modifier that promotes susceptibility to depression, and depression and various interventions prime larger cytokine responses (34, 35). These findings demonstrate the complex pathophysiology of MDD.

To elucidate the molecular intersection of cell senescence with MDD, the expression patterns of cell senescence-related genes between patients with MDD and healthy control participants were analyzed, which revealed the dysregulation of 84 cell senescence-related genes in MDD. After cross-selection using genes associated with MDD disease phenotypes screened using WGCNA, 20 genes related to both MDD and cell senescence were determined. These genes can be considered key genes in the intersection between cell senescence and MDD. Functional enrichment indicated that they were involved in hormone-related signaling pathways (such as the estrogen signaling pathway, response to steroid hormones, and corticosteroids) and immune and inflammatory pathways. Changes in hormone levels and endocrine function have been linked to the pathophysiology of MDD. For example, estrogen mediates brain networks and processes associated with alterations in cognition, emotional dysregulation, and stress responses, which are the main features of MDD (36). Monotherapies or adjunctive strategies based on hormones and hormone-controlling compounds have been investigated for MDD; such therapeutic actions are associated with both the modulation of endocrine systems in the periphery and the CNS effects of hormones on non-endocrine brain circuitry (37).

Next, eight key genes were screened from the 20 genes associated with both MDD and cellular senescence, and these eight genes were downregulated in MDD. In an external independent validation dataset, three genes, JUN, CTSD, and CALR, were downregulated in MDD. Hence, it’s speculated that these three genes are more frequently dysregulated in MDD and may rarely be affected by individual sample differences or sample size, which are important characteristics of predictive biomarkers. However, the involvement of these three genes in the pathophysiology of MDD remains largely unknown. Reportedly, cathepsin D (CTSD) plays a crucial role in myelination of the CNS, and its deficiency can delay myelination, probably by repressing the transport of proteolipid proteins (38); a CTSD-deficient mouse model exhibited depressive-like behaviors under stressful conditions (39). Calreticulin (CALR) is a highly conserved chaperone protein involved in various cellular processes. Mutations in its promoter have been linked to the occurrence of major psychiatric disorders, including MDD (40). In this study, the expression of these three genes strongly correlated with immune cell infiltration in patients with MDD.

Increasing evidence indicate that MDD is accompanied by immune dysregulation (involving both peripheral and brain immune system), which are correlated with each other (41, 42). This reflects the involvement of dysregulated neural-immune interactions in MDD. Specifically, communication of the immune system between the brain and peripheral regions may result in neuroinflammation, thereby affecting neurogenesis and neural plasticity. While neuroinflammation can also initiate dysregulation on cellular immunity, thereby promoting the susceptibility to MDD (34, 43, 44). This study found that all three hub genes (JUN, CTSD, and CALR) were significantly correlated with the abundance of many types of immune cells, such as Tregs, NK cells, and CD4+ T cells, which are crucial immune cells involved in depressive disease progression (45–47). These findings suggest that these three hub genes may be implicated in the immunoinflammatory mechanisms in MDD. Considering the important roles of the three hub genes in MDD, these genes might be potential therapeutic targets for treatment of MDD. Hence, the miRNAs and TFs that target these genes were predicted to explore the possible molecular regulatory mechanisms. Among which, miR-423-5p was both regulated by CALR and CTSD. Liu et al. (48) revealed that miR-423-5p was regulated in MDD group when compared to that in healthy control group. Yoshino et al. (49) found that miR-423-5p showed significant (FDR corrected; p < 0.05) differential regulation in the synaptic fraction from dorsolateral prefrontal cortex of MDD subjects. In addition, JUN and CTSD, particularly JUN, were regulated by multiple TFs, and ESR1, ESR2, and ARNT were TFs that regulated JUN and CTSD. Zhang et al. (50) revealed ESR1 may have therapeutic implications for MDD. Cao et al. (51) suggested the ESR2 was one of the potential risk factors for both MDD and atopic diseases. Thus, these miRNAs and TFs might involve the pathophysiological process of MDD in different aspects. Besides, this study also found that CTSD is a target of streptozocin, and CALR is the target of oltipraz and gentamicin, whereas JUN is a target of more than 40 drugs. A previous study has showed that intracerebroventricular administration of low dose of streptozocin in mouse produces depressive-like behavior (52). However, few studies reported oltipraz and gentamicin on MDD. Thus, small-molecule drugs targeting these three genes were predicted to contribute to the development of novel drug development strategies.

However, this study has numerous deficiencies. Firstly, the dataset included in this study had small sample size, and the datasets with large sample size should be further included. DIANA-TarBase v7.0 should be conducted to validate the concordance with the potential miRNAs regulating the hub target genes identified by ENCORI. In addition, the data analyzed in this study were obtained from public database, and the reliability and robustness of the results should be confirmed in the future. Moreover, the identified immune cells should be tested by flow cytometry. Finally, the hub genes, miRNAs and TFs should be further tested through experimental analyses.

In summary, three cell senescence-related genes were identified as potential diagnostic biomarkers and therapeutic targets for MDD, emphasizing the molecular intersection between cell senescence and MDD. The association between immune dysregulation and MDD crosses a fault line in the conventional diagnostic categorization of MDD. The expression of these three genes were significantly correlated with the abundance of multiple types of immune cells, highlighting the novelty of these biomarkers. Overall, the findings from this study not only extend our knowledge concerning cell senescence-related genes in patients with MDD but also help us to understand the pathophysiology of MDD more comprehensively.

## Data availability statement

Publicly available datasets were analyzed in this study. This data can be found here: Gene Expression Omnibus (GEO) database, accession numbers GSE201332, GSE201332 and GSE52790.

## Ethics statement

The studies involving humans were approved by Ethics Committee of the Chinese Academy of Medical Sciences and the Peking Union Medical College. The studies were conducted in accordance with the local legislation and institutional requirements. Written informed consent for participation in this study was provided by the participants’ legal guardians/next of kin.

## Author contributions

JC: Validation, Formal analysis, Visualization, Writing – original draft. XX: Formal analysis, Validation, Supervision, Writing – review & editing. ML: Validation, Methodology, Writing – original draft. HH: Validation, Investigation, Writing – original draft. TW: Validation, Investigation, Writing – original draft. QL: Validation, Conceptualization, Writing – review & editing. RH: Investigation, Validation, Conceptualization, Funding acquisition, Writing – review & editing.
