# Supplementary material for: Genes associated with cellular senescence as diagnostic markers of major depressive disorder and their correlations with immune infiltration
Source: Front Psychiatry. 2024 May 31;15:1372386. doi: 10.3389/fpsyt.2024.1372386 (PMC11179437; doi:10.3389/fpsyt.2024.1372386)

Supplemental Information

Supplementary Figure 1. Standard curves of measured absorbance at 485 nm for a serial dilution of INT respiratory stain. Three, 12-point calibration curves were generated from 0.06 μmol L^{-1} to 120 μmol L^{-1} INT. The mean slope of the standards (0.0174 µM absorbance units per µM INT) is used to calculate the INT-based respiratory rate of the N. Atlantic seawater samples using the relationship determined by Garcia-Martin et al. (2019a).

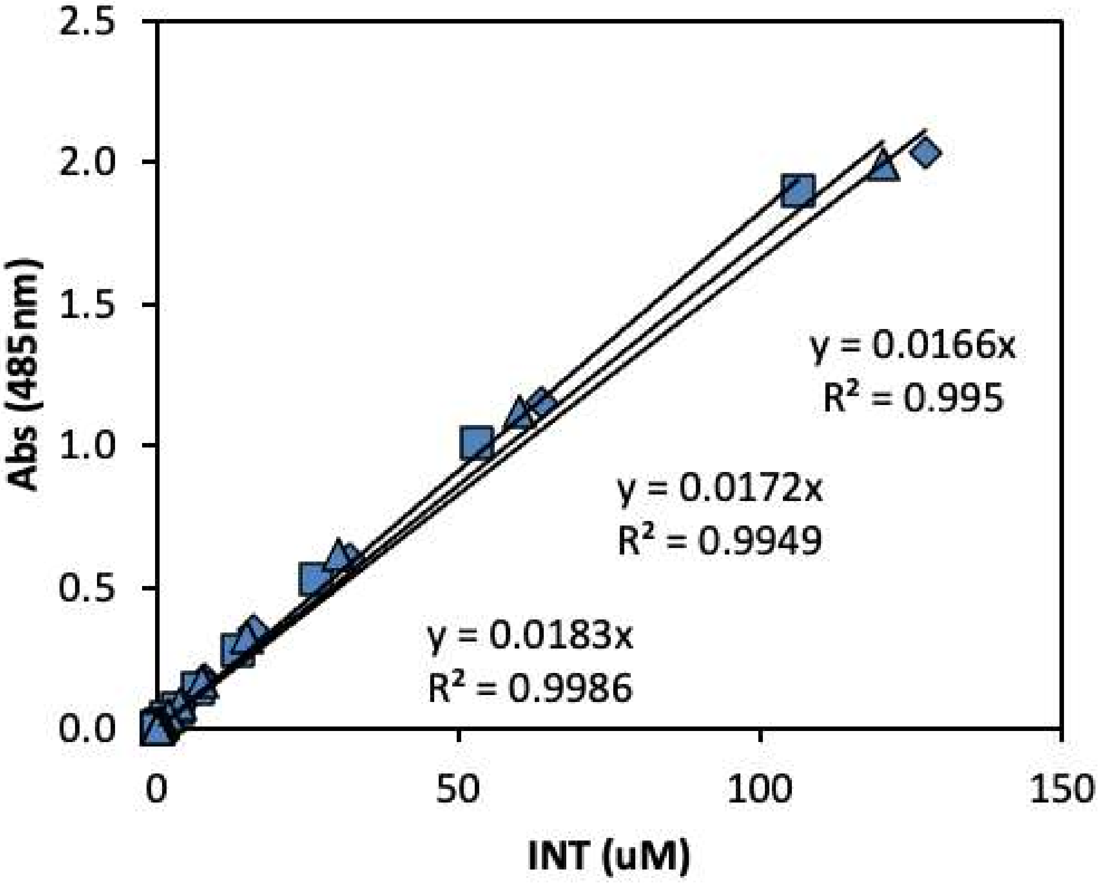

Supplementary Figure 2. Individual N. Pacific respiration assays grouped by sampling day, depth, and size fraction from the using PreSens oxygen sensor spots manually measured every few hours. The respiration rate is the absolute value of the linear regression slope (black line) of measured oxygen concentrations (black points). Assays with rates below detection or with nonlinear rates are indicated on the plot with the label "Exclude".

North Pacific Oxygen Consumption Assays: 2018

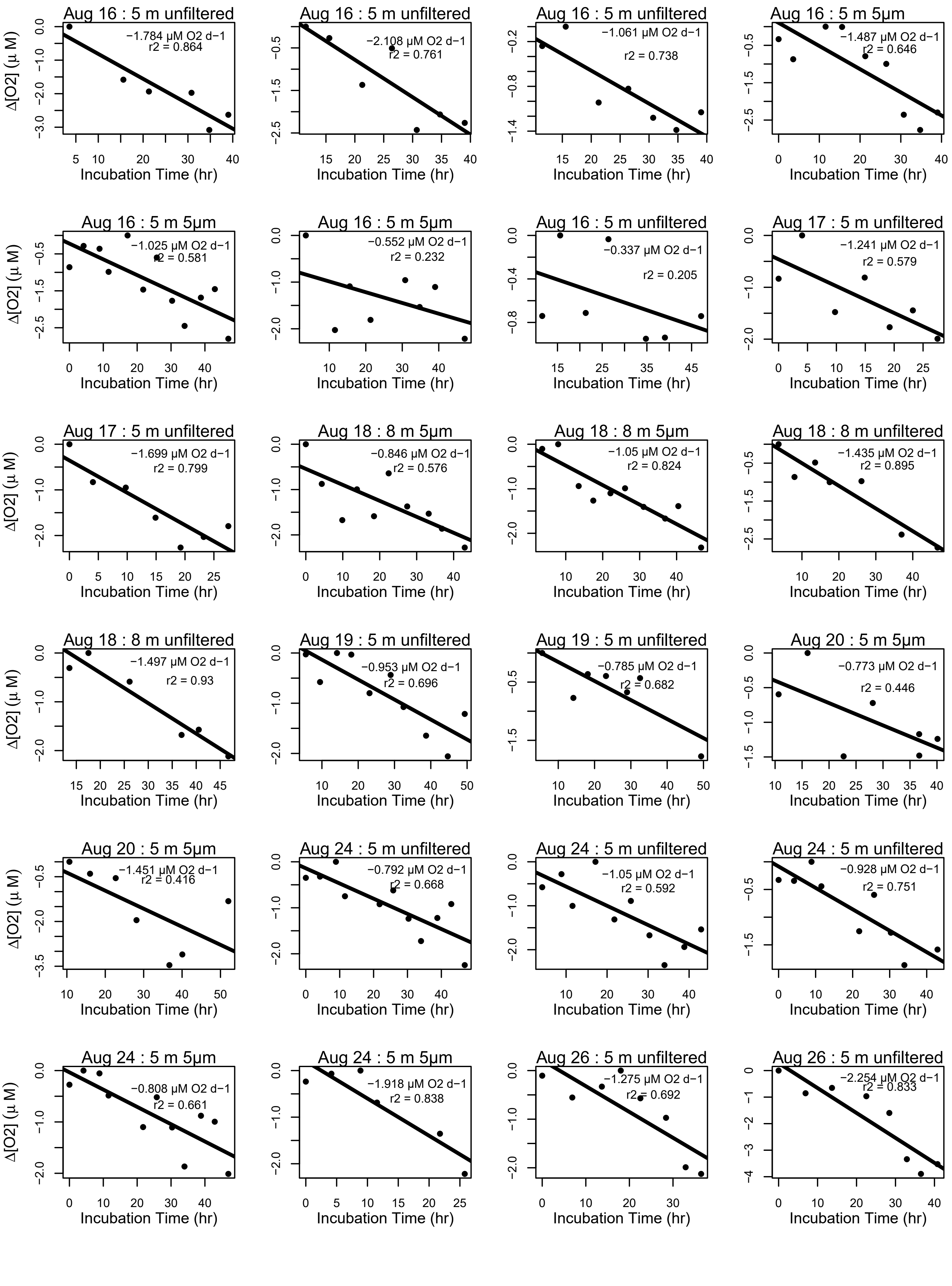

North Pacific Oxygen Consumption Assays: 2018

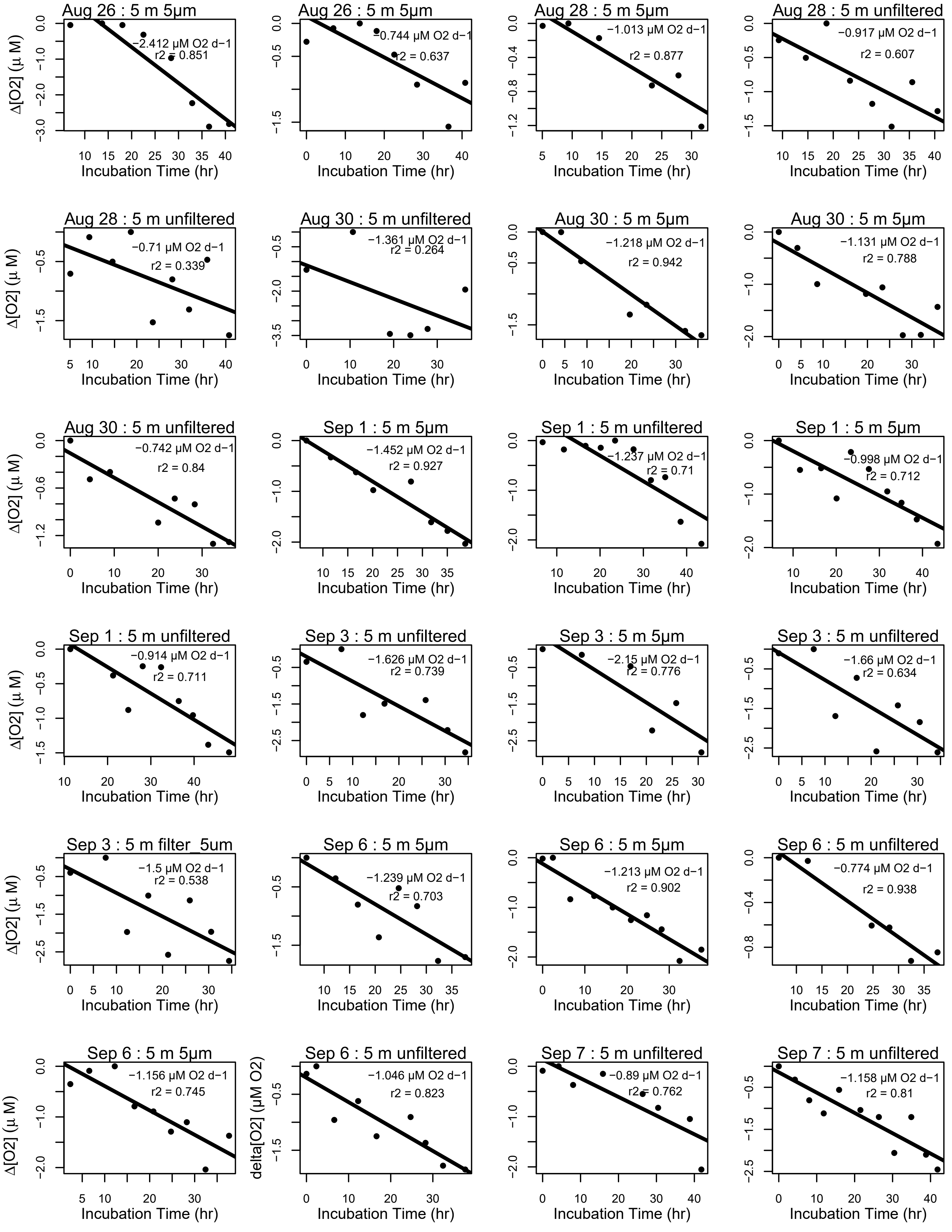

North Pacific Oxygen Consumption Assays: 2018

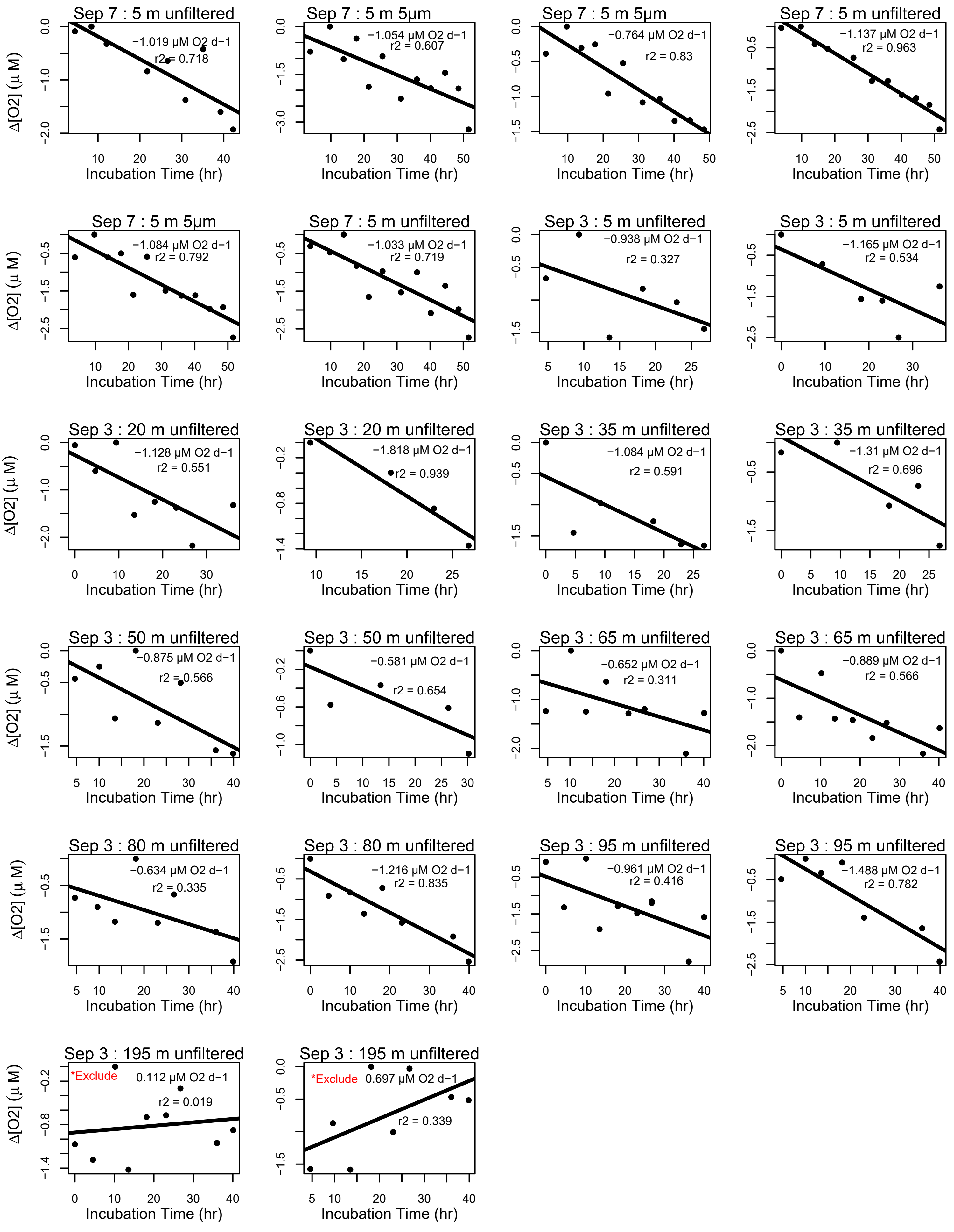

Supplementary Figure 3. Individual N. Atlantic respiration assays grouped by sampling day, depth, and size fraction using continuously mounted fiber optic cables to PreSens oxygen sensor spots. Oxygen concentrations were recorded every 30 s (black points) with removed measurements indicated (gray points). The rolling mean (blue points) and linear regression (red line, statistics in top right corners) show the respiration rate. Assays with rates below detection or with nonlinear rates are indicated on the plot with the label “Exclude”.

North Atlantic Oxygen Consumption Assays: May 06, 2021

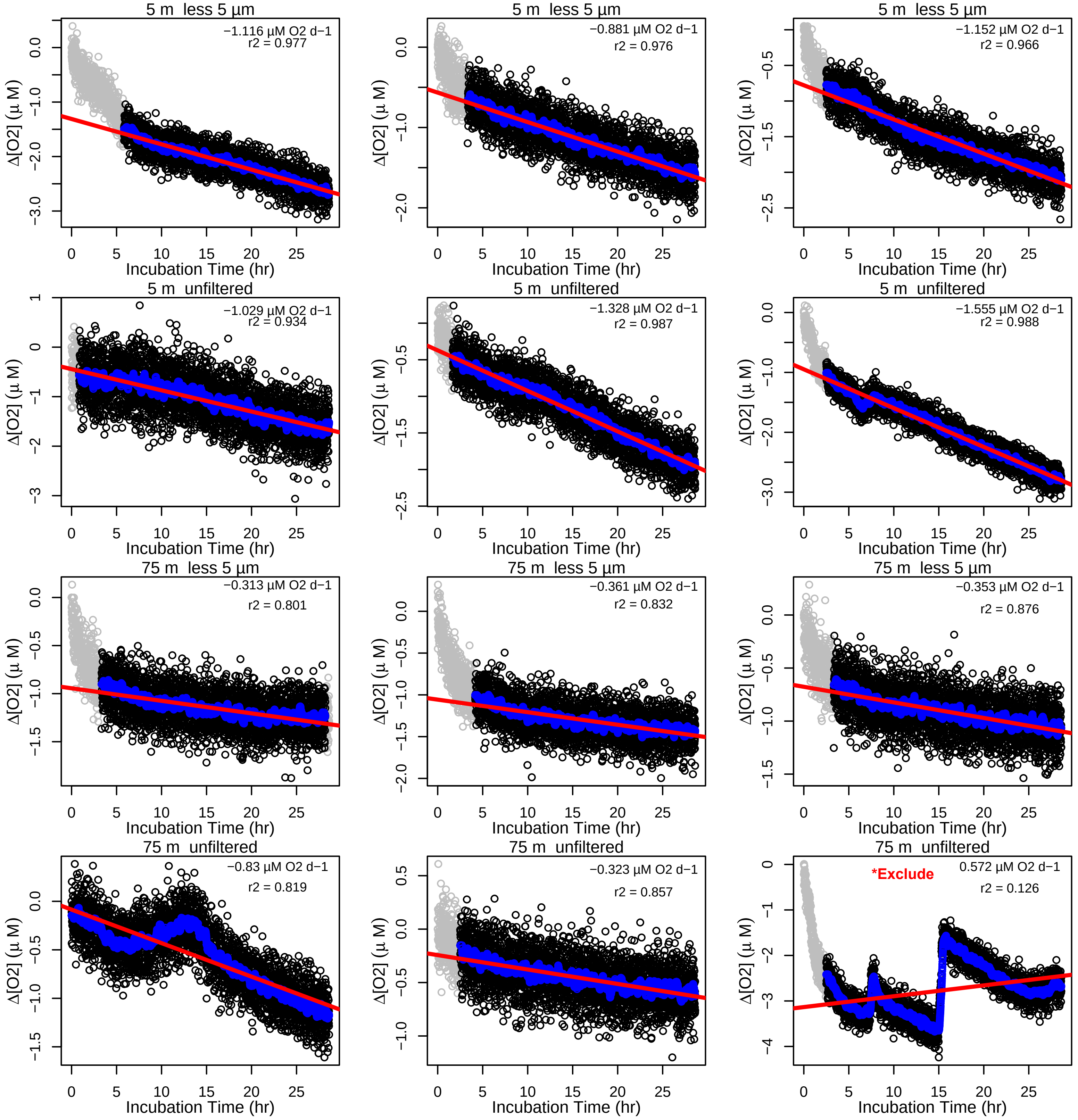

North Atlantic Oxygen Consumption Assays: May 12, 2021

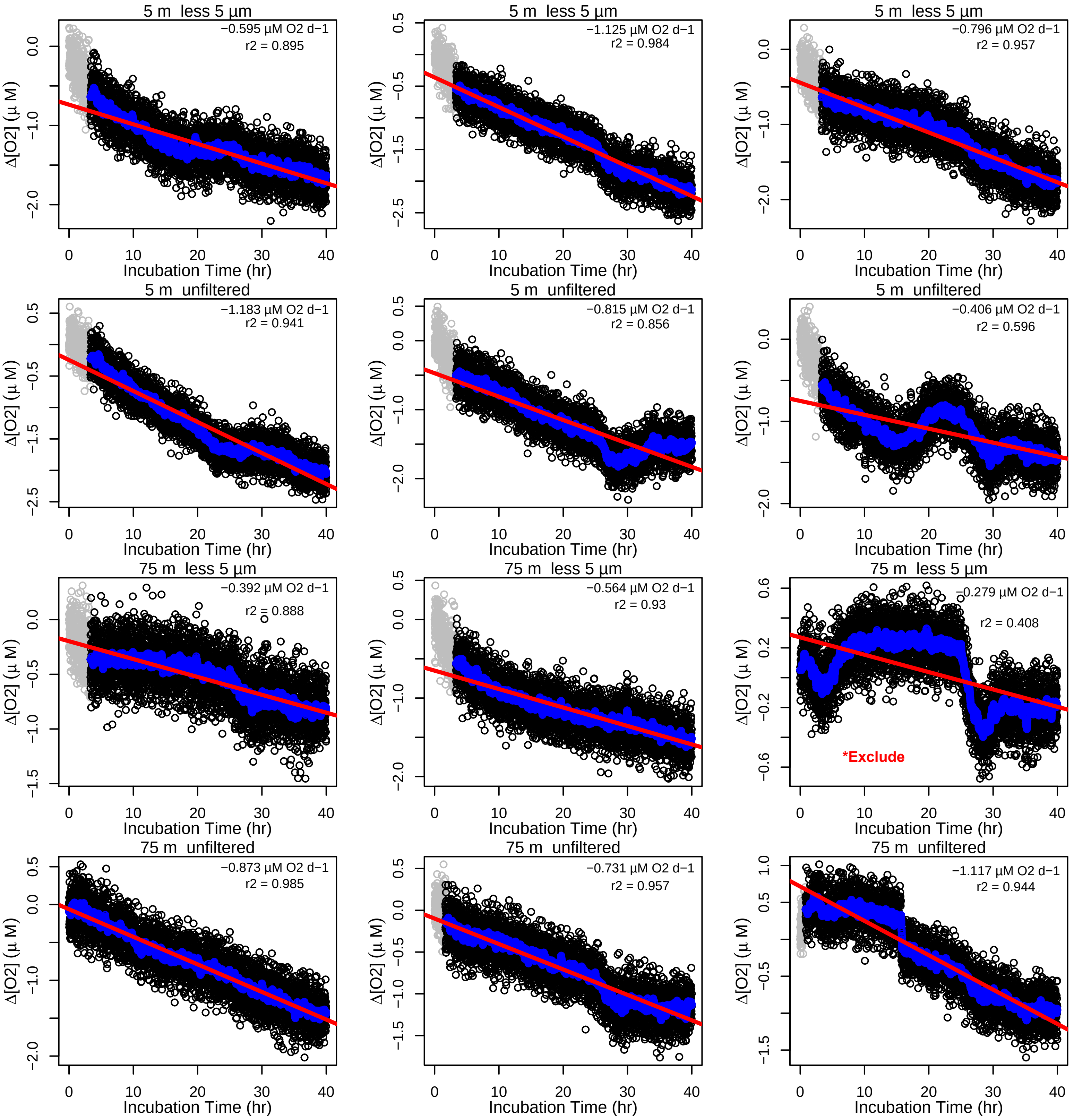

North Atlantic Oxygen Consumption Assays: May 14, 2021

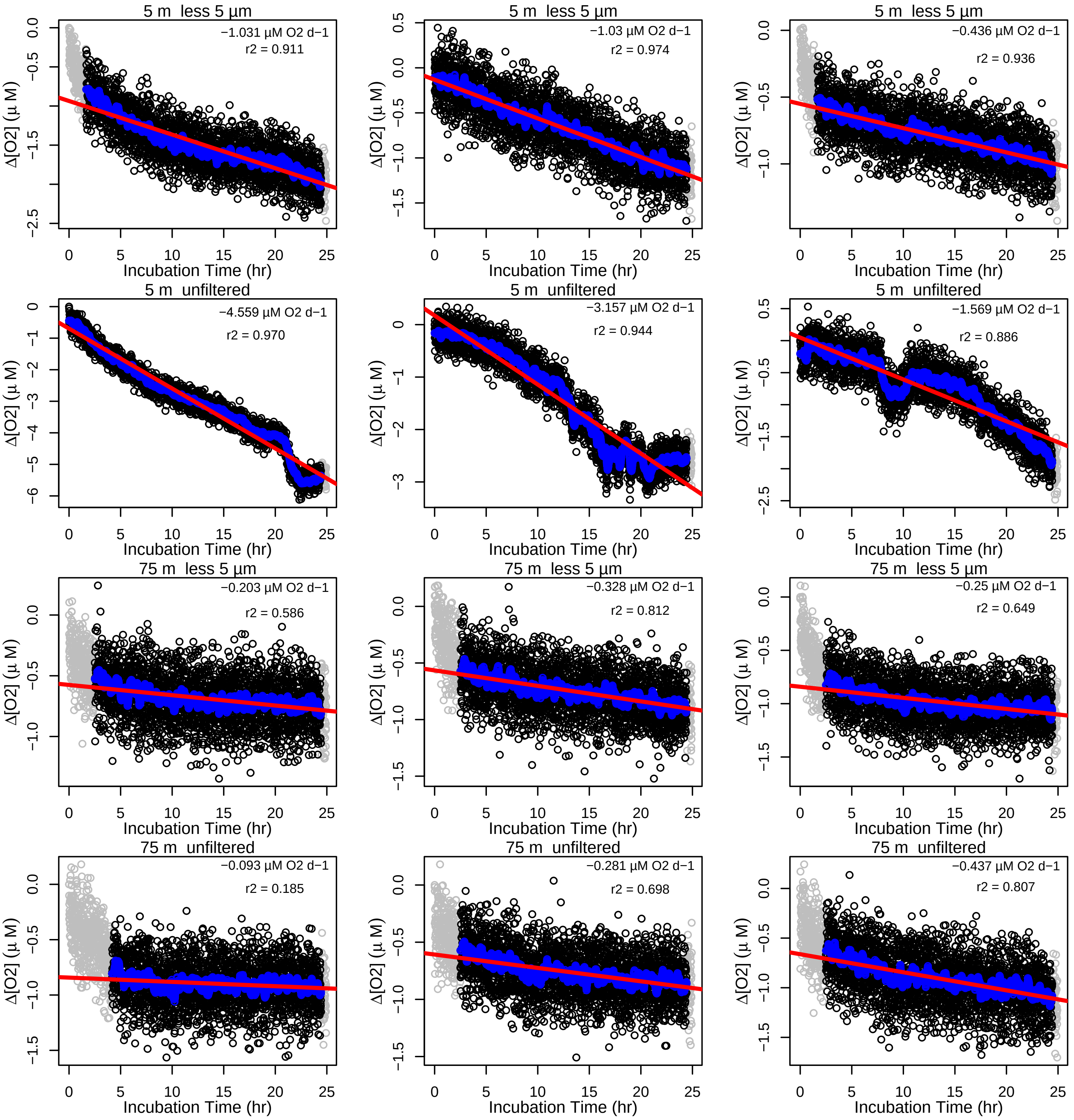

North Atlantic Oxygen Consumption Assays: May 18, 2021

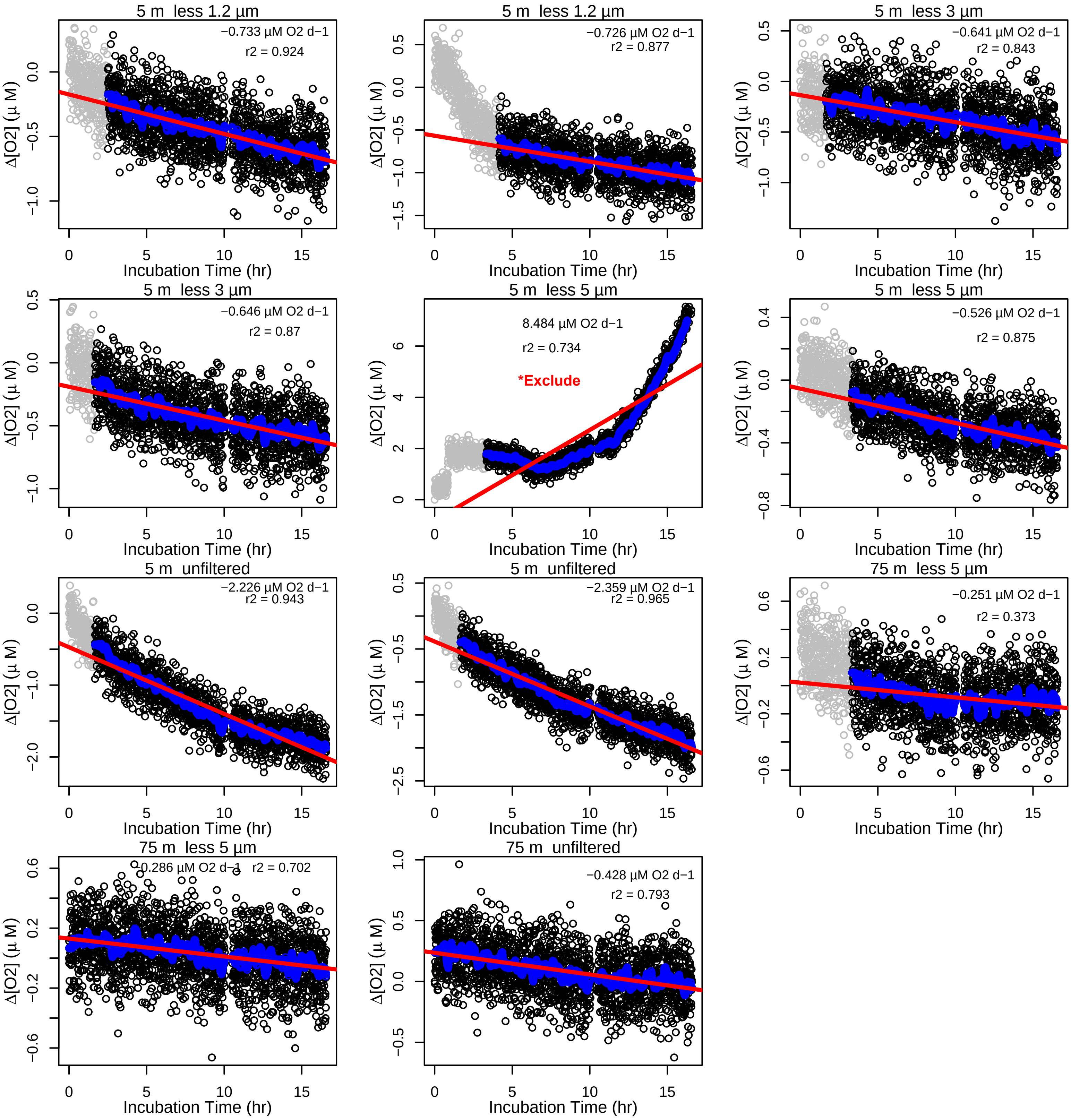

North Atlantic Oxygen Consumption Assays: May 19, 2021

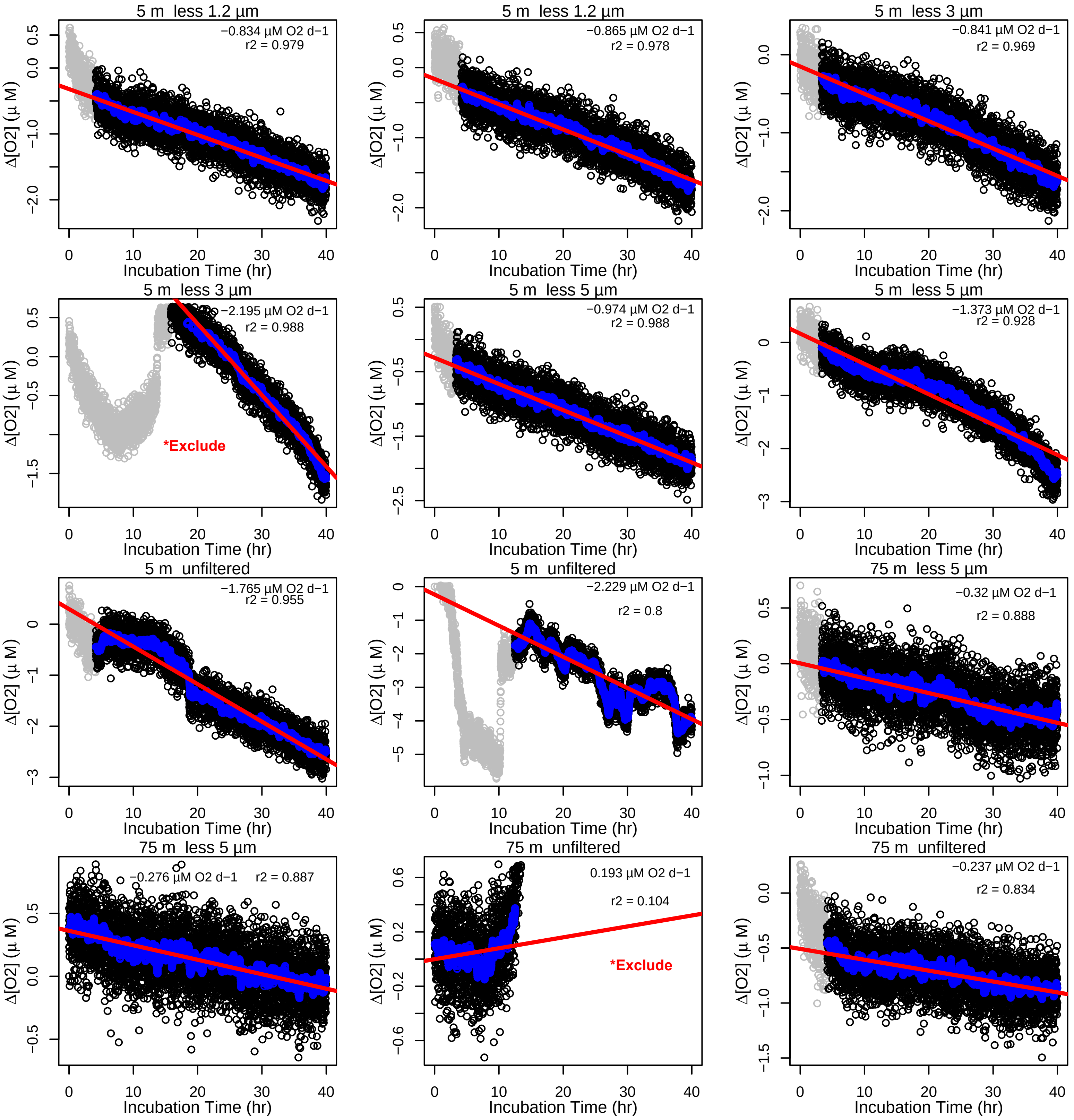

North Atlantic Oxygen Consumption Assays: May 22, 2021

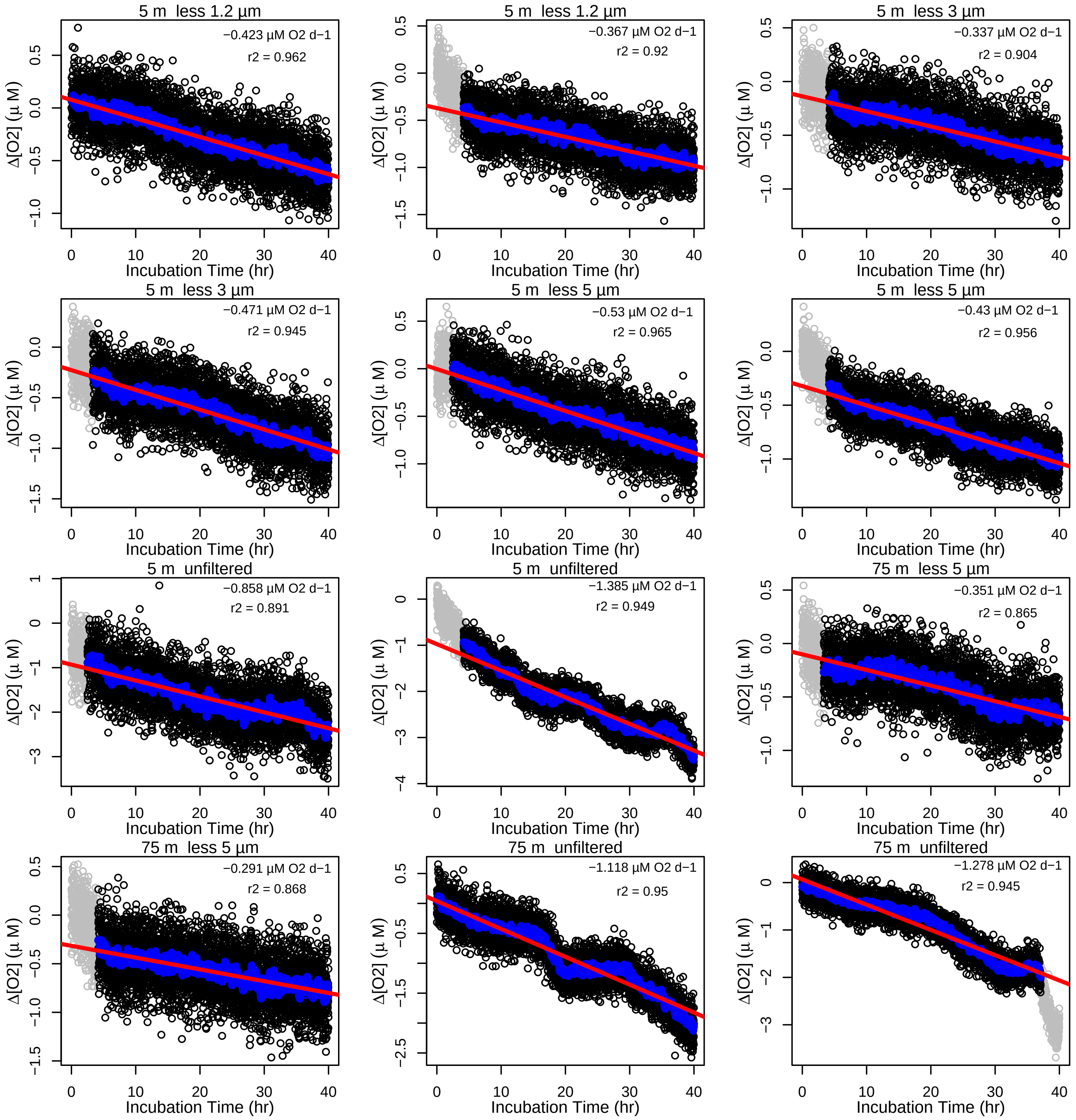

North Atlantic Oxygen Consumption Assays: May 25, 2021

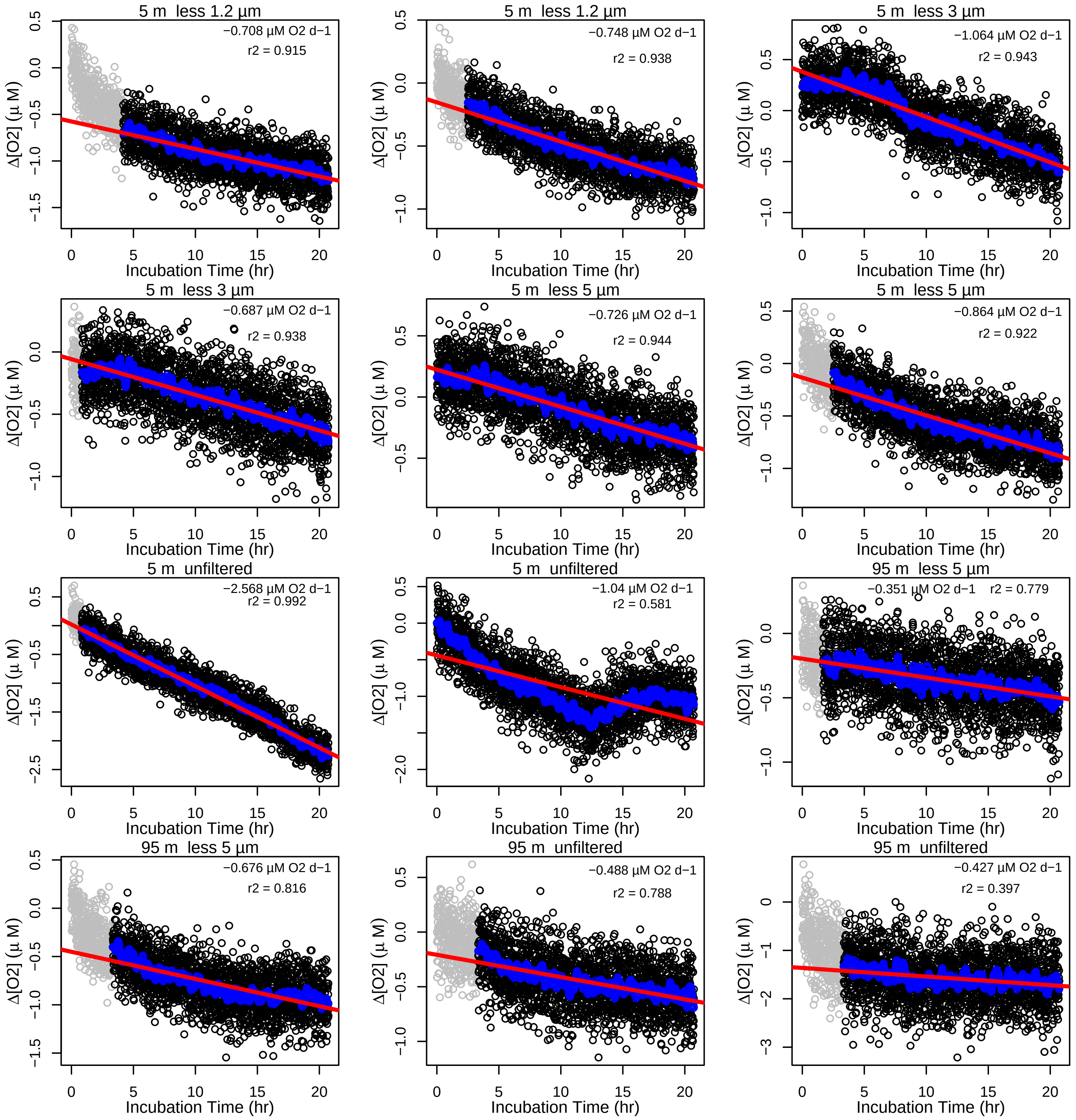

North Atlantic Oxygen Consumption Assays: May 26, 2021

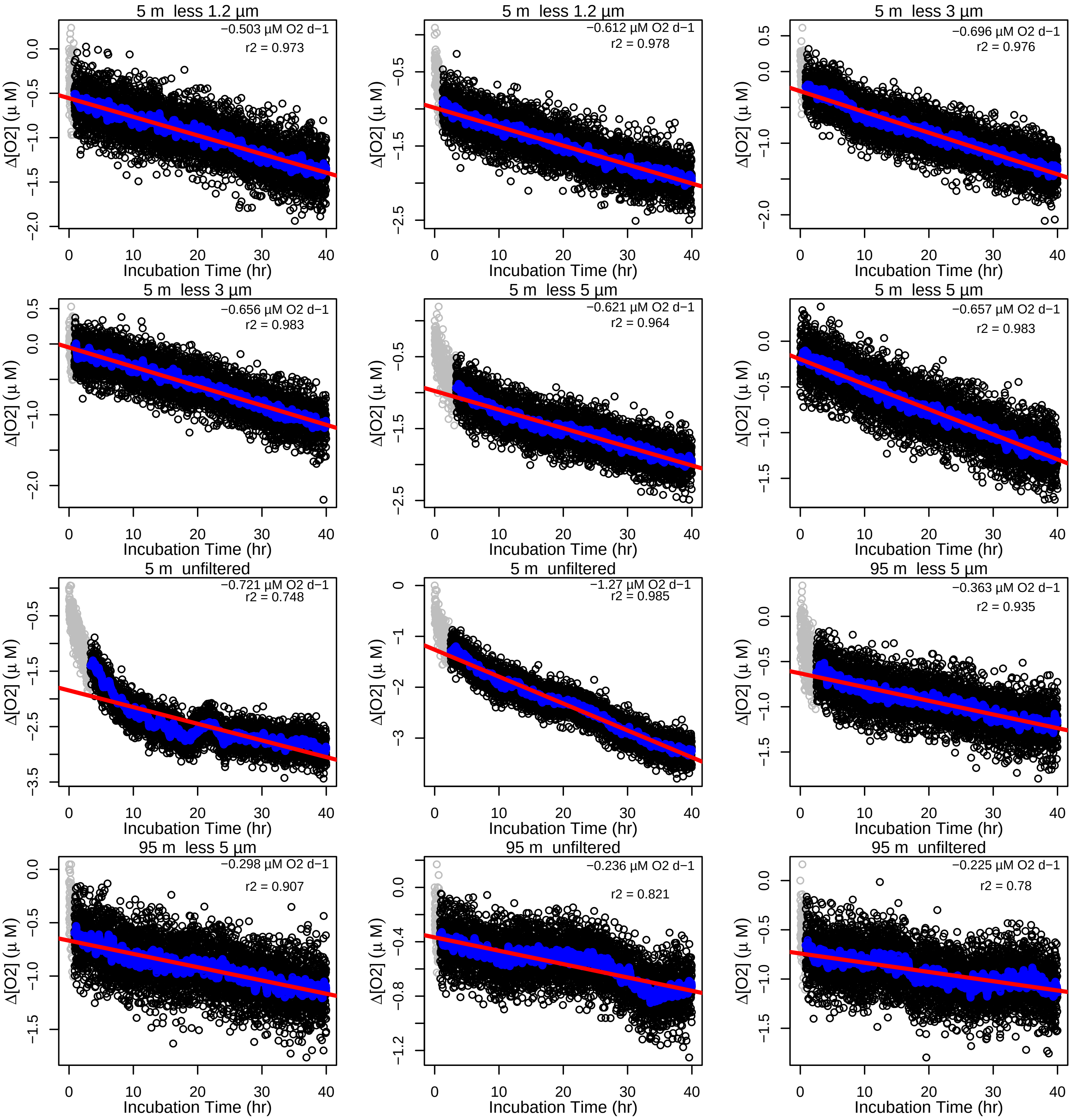

North Atlantic Oxygen Consumption Assays: May 28, 2021

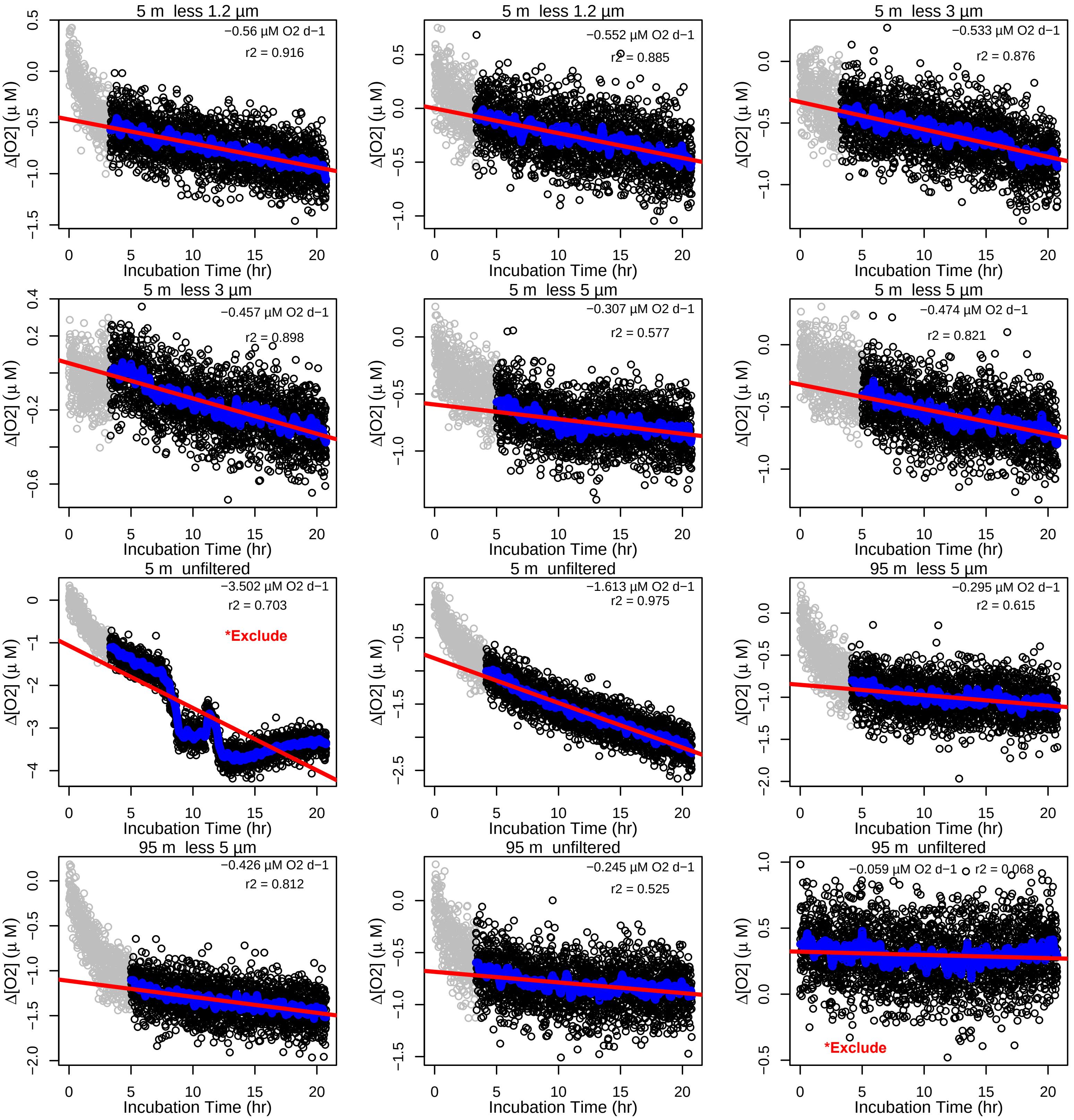

North Atlantic Oxygen Consumption Assays: May 29, 2021

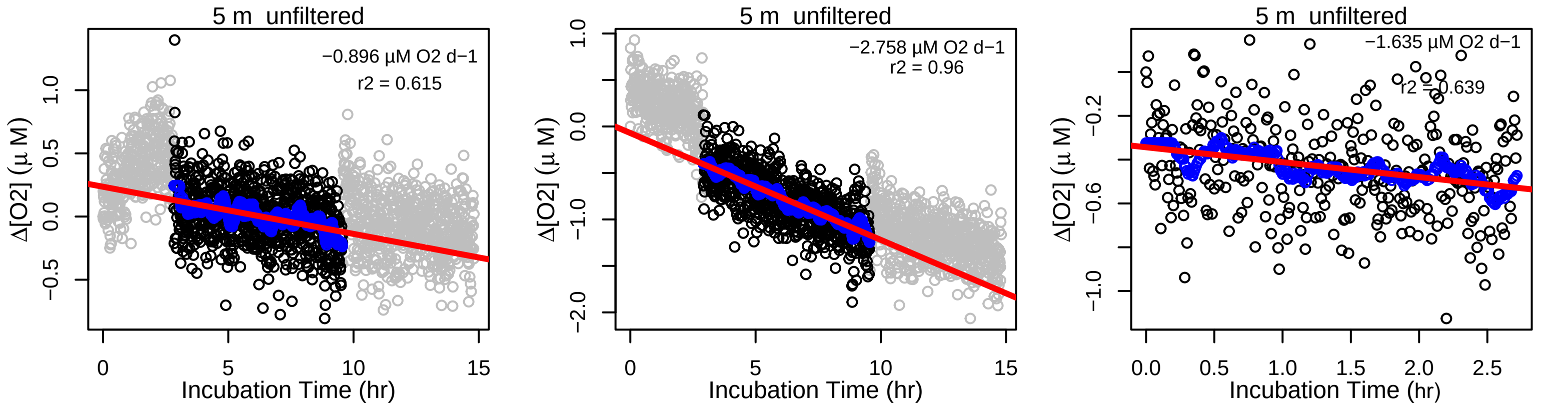

Supplementary Figure 4. Residual standard errors (RSE) of the trimmed N. Atlantic respiration rates. Gray circles indicate the residual standard error for each N. Atlantic assay. The black bars show the mean and 95% confidence interval of the RSEs for each depth treatment. There is no significant difference between the RSE based on depth.

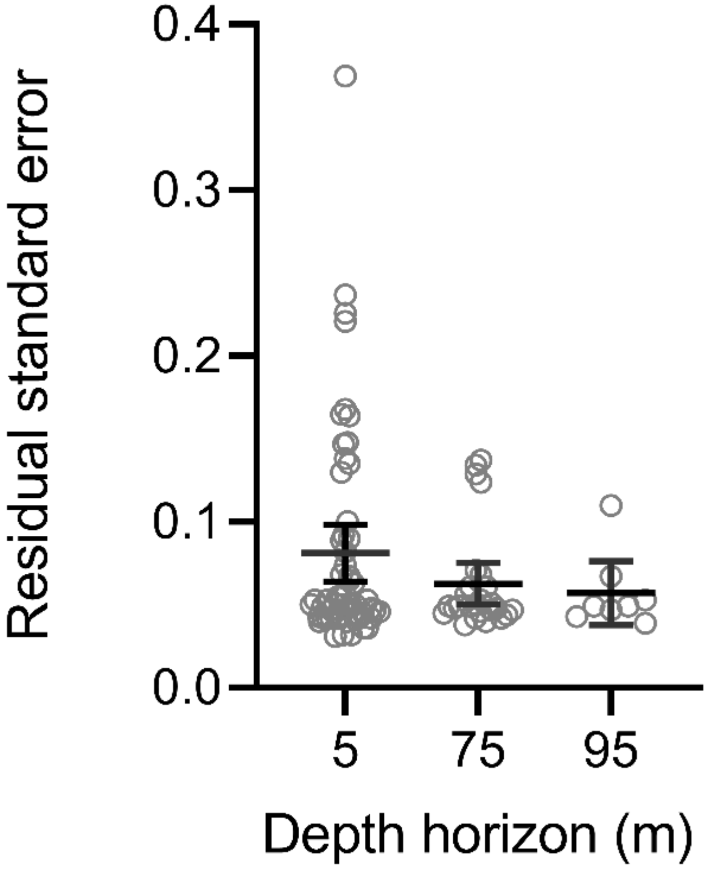

Supplementary Figure 5. Residual Standard Error (N. Atlantic) by size fraction. Horizontal bars indicate the mean RSE within each size class and depth horizon. One outlier was identified in the <5 μm surface fraction using the ROUT method (Q=1%) and removed. No outliers were identified for the <1.2 μm fraction (n=12), the <3 μm fraction (n=9), the <5 μm deep fraction (n=18), or the unfiltered surface and deep fractions (n=18 and n=16). Means of each fraction were compared using a Brown-Forsythe or Welch ANOVA with significant differences among means at p<0.001 indicated (**).

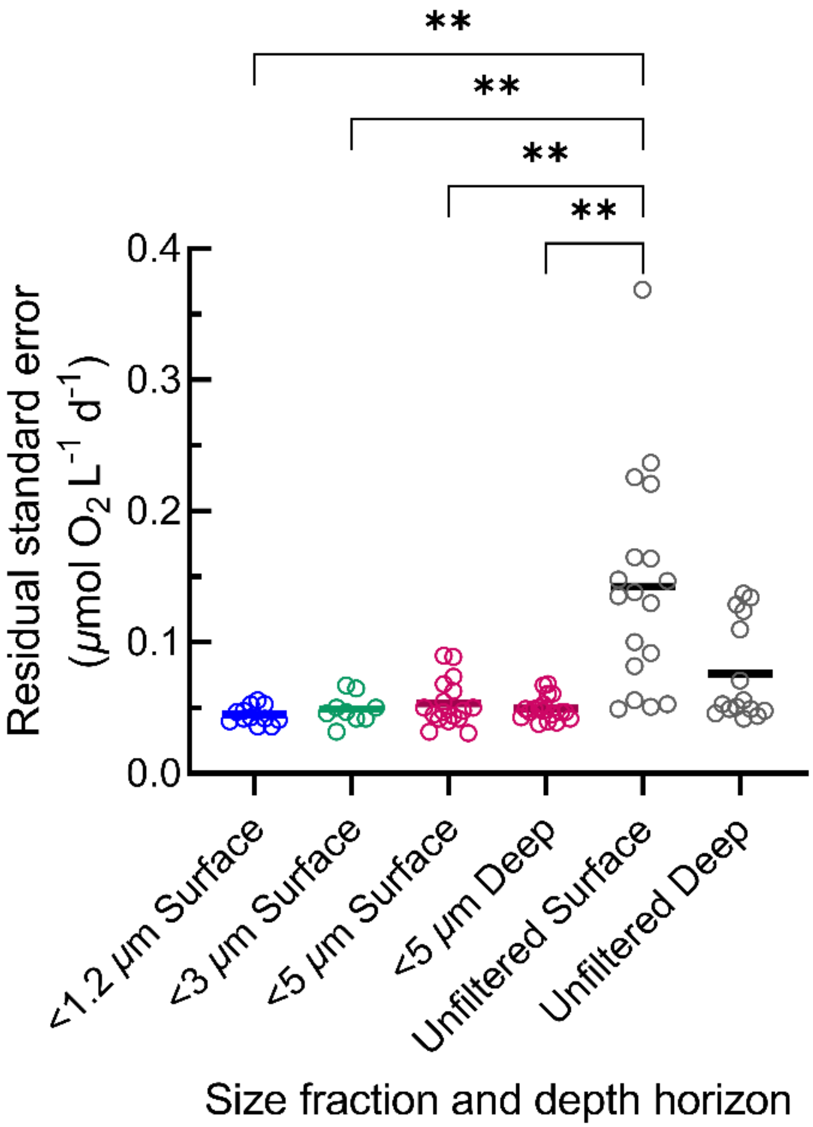

Supplementary Figure 6. Matrix of Pearson’s correlation coefficients (bottom diagonal) and p-values (top diagonal) to identify statistically significant relationships ($p<0.05$, gray shaded cells) between biological variables in the N. Atlantic.

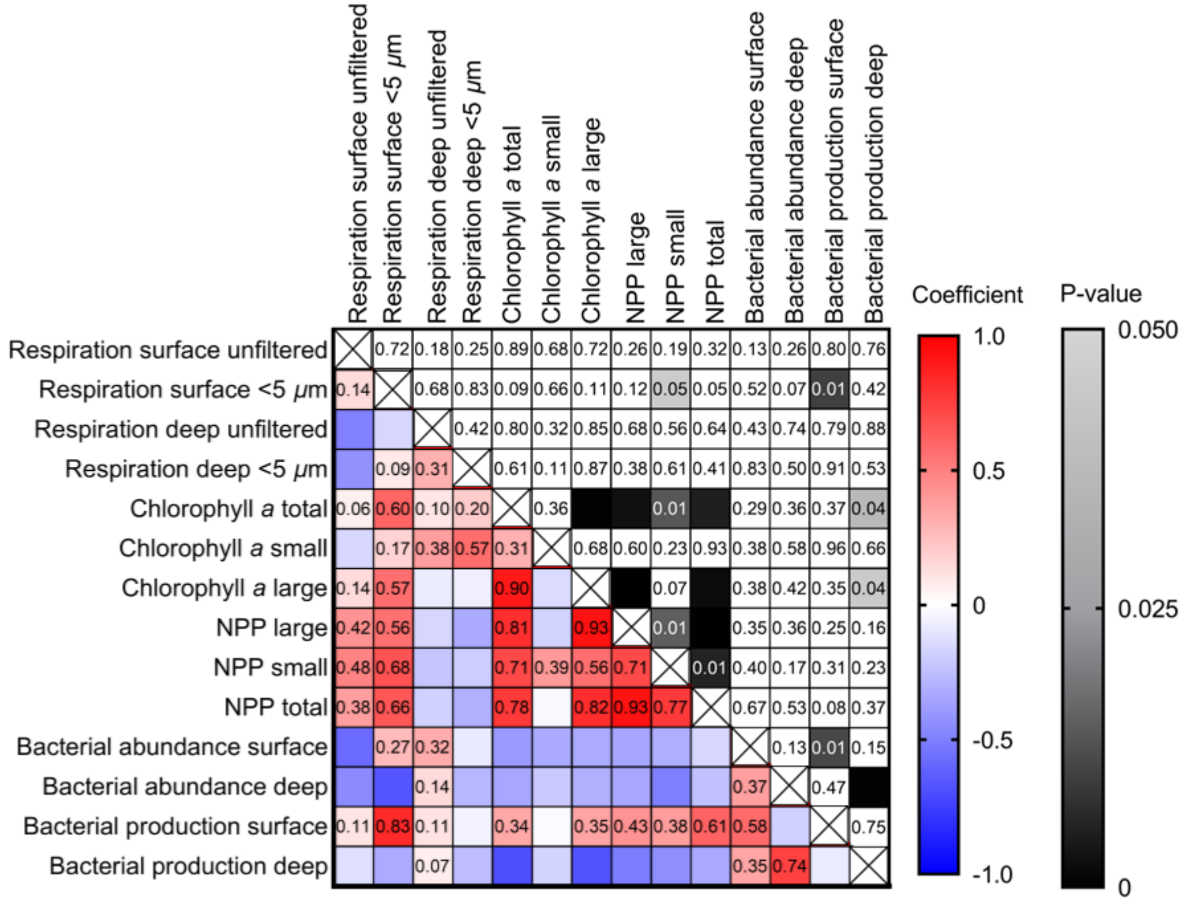

Supplementary Figure 7. Respiration incubation bottle setup from the N. Atlantic assays. Biological oxygen demand (BOD) bottles are filled with no headspace with sample water. A mount holds the polymer optical fiber (POF) to the exterior of the BOD wall such that the POF is aligned with the PreSens PSt3 foil sensor spot (pink) inside the BOD bottle. The holder has set screws which are gently hand-tightened to secure the POF. The base of the holder has rubber grip to prevent slipping when secured to the BOD bottle with a Velcro wrap. BOD bottles are placed in an opaque, temperature—controlled water bath with the sensor spot facing upwards as on the left. The bath is sealed and covered with a black bag with the POFs fed through a small hole and run to the controller boxes and computer. (POF Holder design available at https://github.com/2mrcohn/pub).

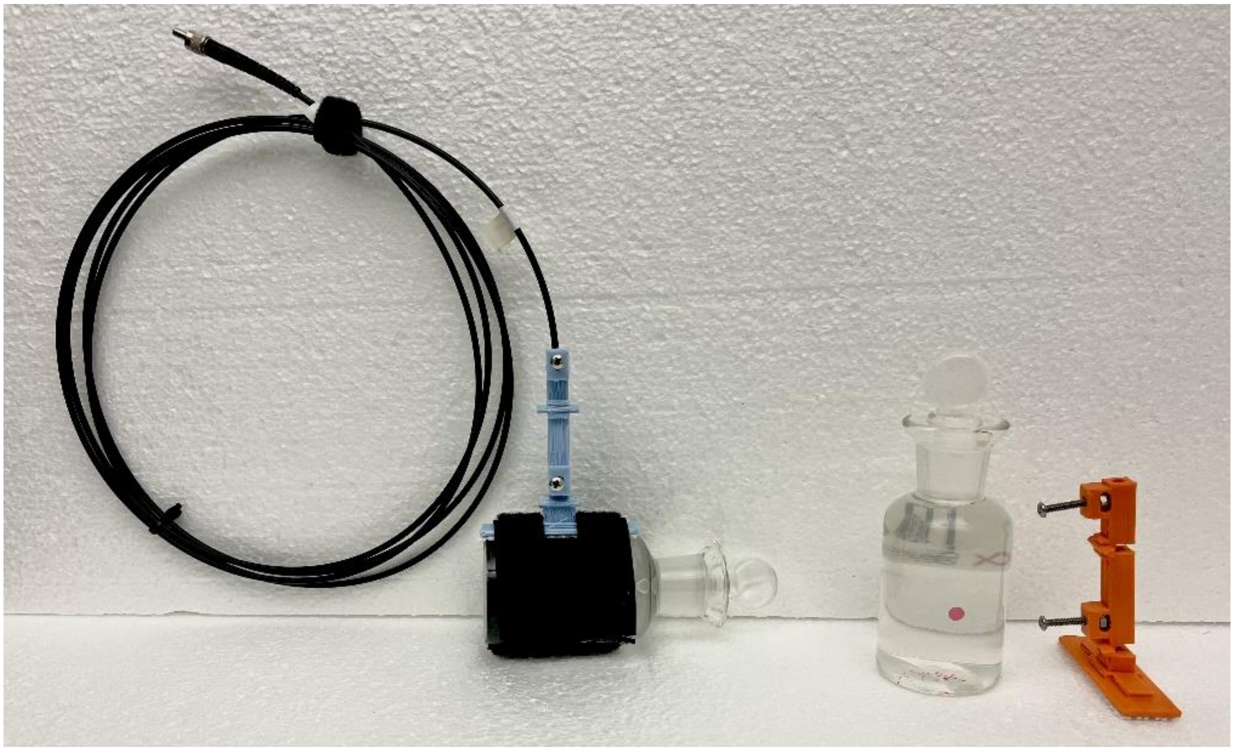

Supplementary Figure 8. The amount of time where O_2 measurements were excluded from plotting as characterized by a non-linear O_2 slope, attributed to sensor and sample temperature acclimation. Based on Milli-Q controls and initial inspection of the assays, 2.5 h were automatically removed from the start of the assay. Additional trimming was determined by visual inspection. The trimming times are grouped by treatment (size fraction and depth). The boxes show the 1st and 3rd quartiles of the time removed from the front of each individual N. Atlantic assay (black points). The horizontal line is the mean and whiskers are drawn if there are points that exceed 1.5 times the interquartile range.

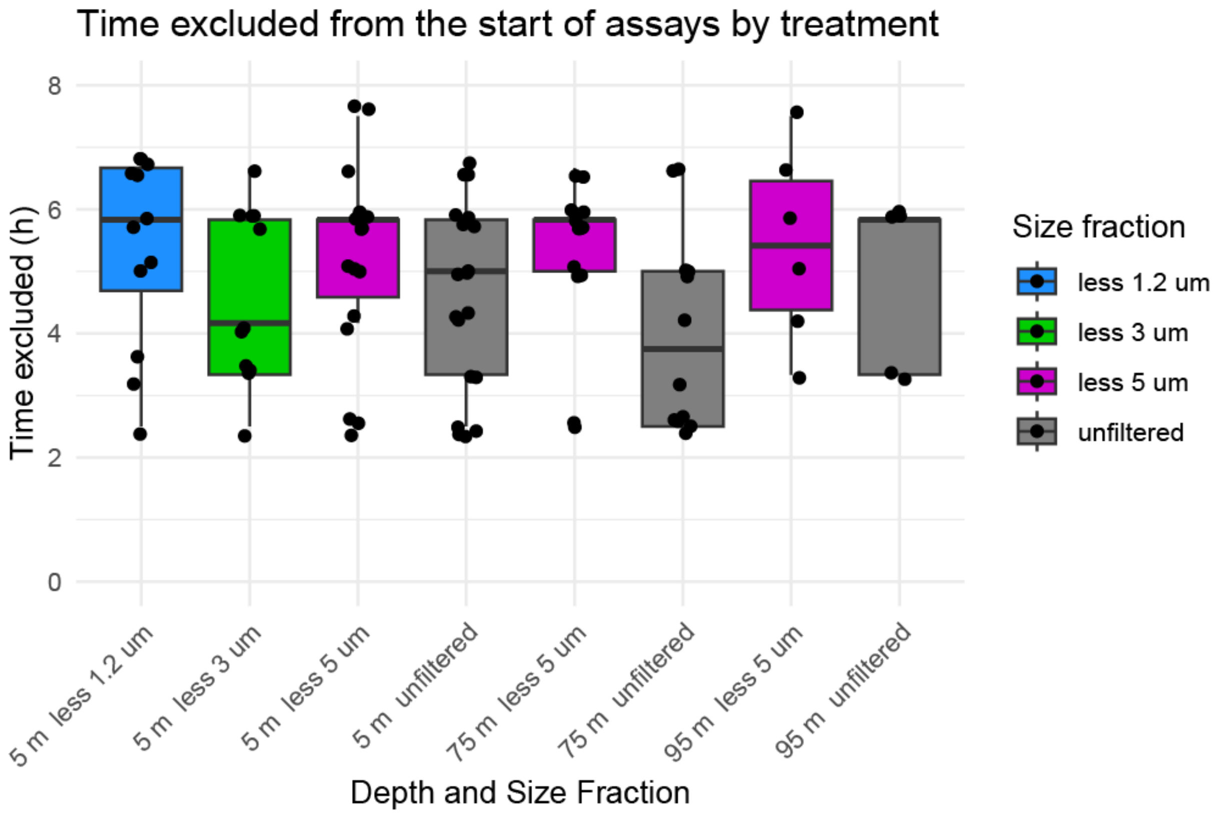

Supplementary Figure 9. The acclimation of the sensor spot from storage to water bath conditions is demonstrated by the apparent O_2 measurements of the Milli-Q control when it was first placed in the bath. The [O_2] readings (open black circles) decrease c.a. 10 µM over the first 2.5 h of incubation in an exponential decay manner. The temperature of the water bath (closed red circles) decreases approximately linearly 0.3°C as the temperature control unit re-establishes *in situ* temperature after having the bath open and adding 13 sample bottles.

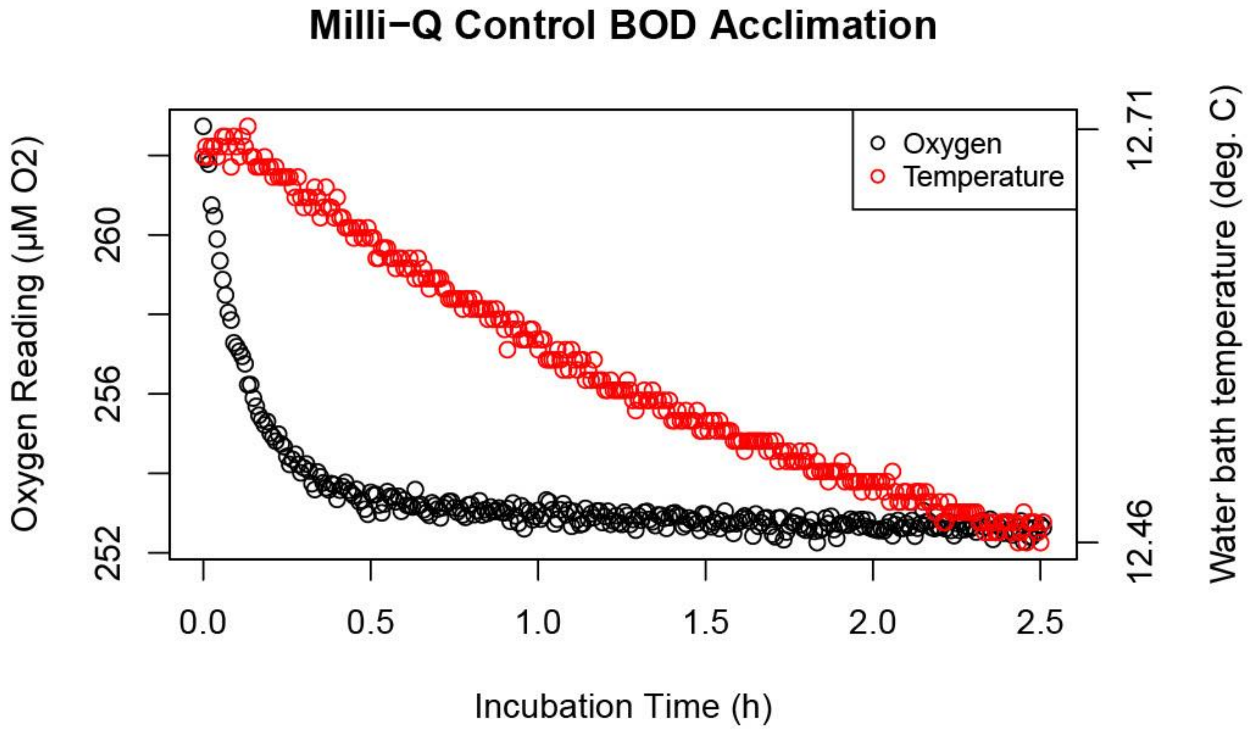

Supplementary Figure 10. Total organic carbon (TOC) concentrations of select respiration incubations compared to CTD rosette casts in the N. Atlantic. Error bars indicate sample supplicates on May 18th and instrumental error on May 25th.

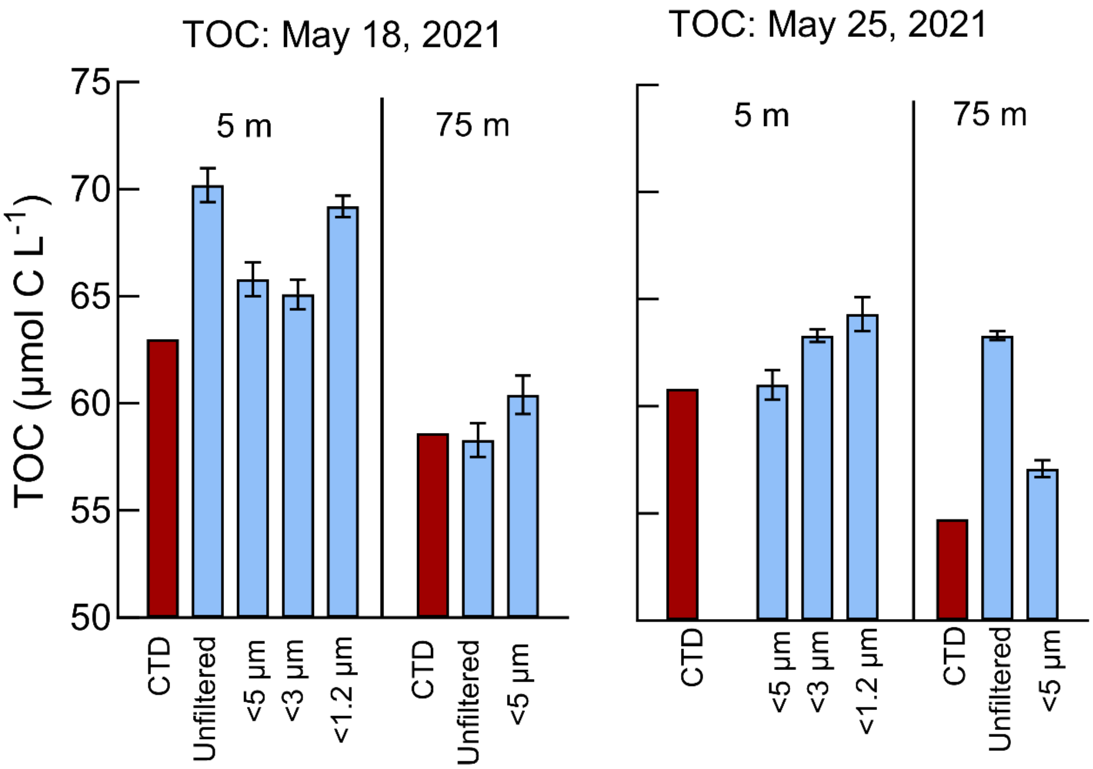

Supplement: Supplementary file 1 [file DataSheet_1.pdf]
